# Management of small cell lung cancer complicated with paraneoplastic Cushing’s syndrome: a systematic literature review

**DOI:** 10.3389/fendo.2023.1177125

**Published:** 2023-10-17

**Authors:** Yanlong Li, Caiyu Li, Xiangjun Qi, Ling Yu, Lizhu Lin

**Affiliations:** ^1^ The First Clinical School of Guangzhou University of Chinese Medicine, Guangzhou, China; ^2^ The First Affiliated Hospital of Guangzhou University of Chinese Medicine, Guangzhou, China

**Keywords:** small cell lung cancer, paraneoplastic Cushing’s syndrome, neuroendocrine tumor, management, immunohistochemistry, infection

## Abstract

Paraneoplastic Cushing’s syndrome (PCS) is a rare, but clinically important feature of small cell lung cancer (SCLC) that is associated with even worse prognosis. To identify key considerations in comprehensive management of SCLC patients complicated with PCS, we conducted a systematic review of relevant reports on PubMed and Web of Science, focusing on SCLC with PCS cases. The systematic review analyzed 61 reports published between 1985 and 2022 with a total of 157 SCLC patients included. Out of the 157 patients, 132 (84.1%) patients across 58 (95.1%) reports were diagnosed with ectopic Cushing’s syndrome. The immunohistochemical (IHC) staining for adrenocorticotropic hormone (ACTH) was performed on 30 (19.1%) patients across 22 (36.1%) reports and demonstrated encouraging performance. For treatment, chemotherapy and ketoconazole were utilized in 50 (81.97%) and 24 (39.34%) reports, respectively. Regarding cause of death, infection and cancer were equally frequent, each being recorded in 17 (27.87%) reports. To conclude, the majority of PCS cases in SCLC patients were caused by ectopic hormone secretion. In order to make a differential diagnosis, it is recommended to utilize IHC staining for a specific hormone such as ACTH or corticotropin-releasing hormone. In the comprehensive treatment of SCLC with PCS patients, effective management of hypercortisolism and potent safeguarding against infection play two crucial roles. Ultimately, further confirmations are required regarding the specificity and accuracy of IHC staining technique as well as the efficacy and safety of immunotherapy in the treatment of SCLC with PCS patients.

## 1 Introduction

Small cell lung cancer (SCLC), a highly aggressive subtype of lung cancer, is characterized by rapid proliferation, high growth fraction, and early development of metastases. It accounts for approximately 14% of all lung cancer cases and possesses a particularly poor prognosis (1, 2). Likewise, ectopic Cushing’s syndrome (ECS), hypercortisolism due to ectopic hormone secretion, is estimated to account for 5%–10% of all Cushing’s syndrome (CS) cases (3–5). Furthermore, the SCLC patients complicated with paraneoplastic Cushing’s syndrome (PCS), mostly caused by ectopic hormone secretion from tumor tissues, comprise a smaller proportion [reported as 1.6%–6% of all SCLC cases (6–9)] but possess an even poorer prognosis among all SCLC patients (6, 8). Retrospective studies have shown that median survivals of SCLC patients with PCS were less than 7 months (6–11).

Regarding the management of SCLC with PCS patients, although some effective hypercortisolism controlling methods exist (3, 12, 13), there was little advancement in treating SCLC for over three decades before the advent of immune checkpoint inhibitors (ICIs) modestly improved its overall survival (14–16). However, several reports have emerged on immunotherapy-induced CS, drawing much attention to the adverse effect (17–20). Considering PCS being a poor prognosis marker for SCLC patients, early and further differential diagnosis of CS is relevant for evaluating prognosis of SCLC patients.

Although there have been case reports, case series, and retrospective studies on SCLC complicated with PCS, no systematic review has been carried out on this topic before. Accordingly, we conducted one, incorporating relevant reports of SCLC with PCS available on PubMed and Web of Science. Through this review, we aimed to illustrate the treatment status of this disease previously and to identify key considerations for comprehensive treatment of these patients.

## 2 Methods

The systematic review has been conducted and reported following the Preferred Reporting Items for Systematic Reviews and Meta-Analyses (PRISMA) 2020 statement (21).

### 2.1 Literature search and selection

We performed a systematic review of relevant reports of SCLC complicated with PCS on PubMed and Web of Science. The search queries are “*(cushing[Title/Abstract]) AND ((sclc[Title/Abstract]) OR (small cell lung cancer[Title/Abstract]) OR (small cell lung carcinoma[Title/Abstract])) NOT ((nsclc[Title/Abstract]) OR (non small cell lung cancer[Title/Abstract]) OR (non small cell lung carcinoma[Title/Abstract]))*” on PubMed and “*(TS=cushing) AND ((TS=sclc) OR (TS=small cell lung cancer) OR (TS=small cell lung carcinoma)) NOT ((TS=nsclc) OR (TS=non small cell lung cancer) OR (TS=non small cell lung carcinoma))*” on Web of Science, respectively. The literature retrieval was performed on 19 November 2022, without restriction on publication date or language.

Selection criteria were as follows: (1) clinical case or case series, prospective or retrospective study, systematic review, or meta-analysis; (2) special reports on this topic or relevant articles involving management of SCLC with PCS patients; (3) critical data available, at least the information on clinical presentation, therapeutic strategy, or causes of death; and (4) no preference for publication date or language. The study was supplemented by screening references of selected articles.

**Figure 1:**
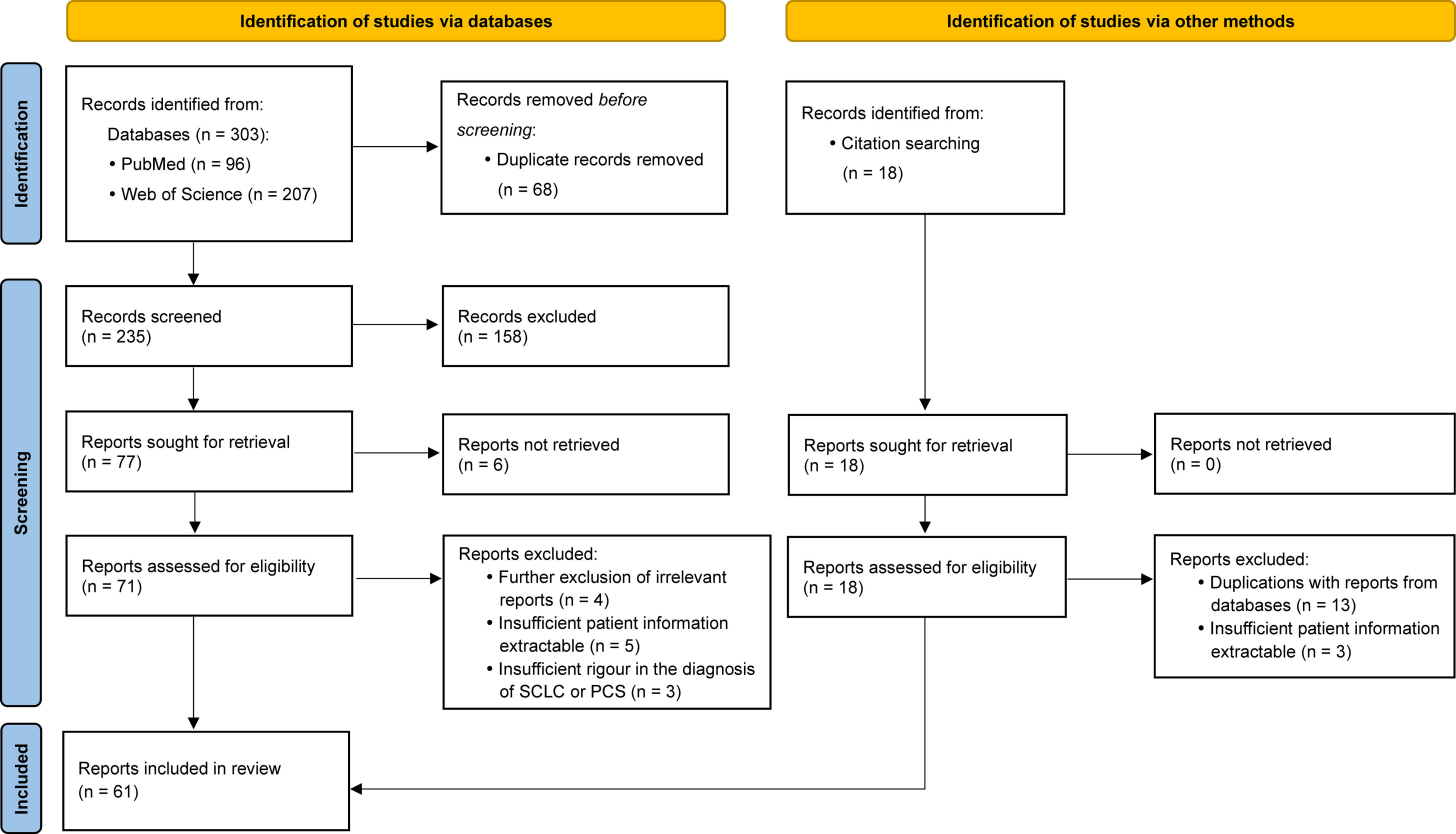
Flow diagram for publication selection procedures performed according to the PRISMA 2020 Statement.

Selection procedures are presented in **Figure 1**. The evidence quality of included reports has been evaluated using the critical appraisal tools provided by the Joanna Briggs Institute (JBI).

### 2.2 Data extraction and analysis

The following information were collected from eligible articles: age, gender, clinical presentation, the immunohistochemical (IHC) staining for adrenocorticotropic hormone (ACTH), therapeutic strategy, survival time/follow-up time, and cause of death. For retrospective and prospective studies included, the quantitative data on age, survival time, and follow-up time were represented as median (minimum–maximum), while the descriptive information on clinical presentation, therapeutic strategy, and cause of death were recorded as percentages wherever possible. As for clinical presentation, therapeutic strategy, and cause of death, we focused on specific details of critical significance for diagnosis and management of the disease. These specific details were categorized and their frequencies were presented.

## 3 Results

Throughout the entire selection process, 61 articles were retrieved, comprising 44 case reports (22–65), 7 case series (66–72), 9 retrospective studies (6–11, 73–75), and 1 prospective study (76) published between 1985 and 2022 in English, Japanese, French, German, Spanish, and Korean. A total of 157 SCLC with PCS patients were involved in these 61 articles. **Table 1** presents details of included reports and patients while **Table 2** displays frequencies of specific details on clinical presentation, therapeutic strategies, and cause of death. The evidence quality of all 61 included reports was evaluated using JBI critical appraisal checklists for case reports, case series, and cohort studies, and results are presented in **Supplementary Materials**.

**Table 1:** The 61 reports of SCLC complicated with PCS from PubMed and Web of Science.

ID	First Author	Year	Journal	Study Design	N	PCS	Age (median)	Gender (M/F)	Clinical Presentation	IHC staining for ACTH	Treatment	Survival (median)	Cause of Death	Follow-up
1(22)	Shepherd	1985	Arch Intern Med	case report	1	ECS^▴^	56	M	1. hypokalemia 6. weakness		1. chemotherapy 4. ketoconazole	5 months	1. tumor progression	
2(23)	Hoffman	1991	Cancer	case report	1	ECS	60	F	1. hypokalemia 5. edema 6. myopathy, weakness 9. polyuria		1. chemotherapy 4. ketoconazole	3 months	3. rapidly increasing ACTH and cortisol levels 6. condition deteriorated	
3(9)	Dimopoulos	1992	Cancer	retrospective	11	ECS	62(49-65)	3/8	1. hypokalemia (100%) 3. glycemia (91%) 4. metabolic alkalosis (100%) 6. weakness (55%) 7. dyspnea (27%)		1. chemotherapy 5. metyrapone	12 days (2-45)	2. infection (73%) 4. cardiac complications 5. respiratory complications 6. miscellaneous, not know	
4(8)	Shepherd	1992	J Clin Oncol	retrospective	23	ECS^▴^	60(43-77)	17/6	1. hypokalemia (96%) 3. hyperglycemia (59%) 4. metabolic alkalosis (96%) 5. edema (83%) 6. muscle weakness (61%)		1. chemotherapy 4. ketoconazole 5. aminoglutethimide	6.23 months (0-20)	1. progressive malignancy 2. infection 5. pneumonia 6. other causes	
5(7)	Delisle	1993	Arch Intern Med	retrospective	14	ECS	62(36-67)	7/7	1. hypokalemia (100%) 2. hypertension (14%) 3. hyperglycemia (71%) 4. metabolic alkalosis 5. peripheral edema (36%) 6. proximal myopathy (29%)		1. chemotherapy 2. radiotherapy	5.5 months (0.75-22)	1. progressive growth of tumor(79%) 2. infections (21%)	
6(67)	Rieu	1993	Horm Res	case series	2	ECS	55	M	2. hypertension 3. hyperglycemia		1. chemotherapy 2. radiotherapy 6. octrotide	2 months	1. metastatic disease	
						ECS	34	F	5. edema 7. dyspnea		1. chemotherapy 2. radiotherapy 3. thoracotomy 4. ketoconazole 5. metyrapone 6. lanreotide, octreotide	8 months	1. metastatic disease	
7(24)	Tabata	1993	Nihon Kyobu Shikkan Gakkai Zasshi	case report	1	CS	62	M	1. hypokalemia 2. hypertension 3. hyperglycemia 4. alkalosis 9. polyuria	(-)	1. chemotherapy	5 months	1. cancer 5. pneumonia	
8(25)	Auchus	1994	J Endocrinol Invest	case report	1	ECS★	75	F	1. hypokalemia 2. hypertension 4. metabolic alkalosis 7. dyspnea	(-)	1. chemotherapy 5. metyrapone	/	/	
9(26)	Huang	1994	Changgeng yi xue za zhi	case report	1	ECS	25	M	1. hypokalemic 3. hyperglycemia 4. alkalosis 7. dyspnea	(+)	4. ketoconazole	13 days	1.progression of lung lesion 2. Nocardia infection	
10(10)	Winquist	1995	J Clin Oncol	retrospective	9+1♦	ECS^▴^	58.5 (44-71)	8/2	1. hypokalemia 2. hypertension 3. diabetes mellitus 4. metabolic alkalosis 5. edema 6. muscle weakness		1. chemotherapy 4. ketoconazole	/	1. progressive cancer 3. hormonal disorder	
11(27)	Takano	1996	Nihon Kyobu Shikkan Gakkai Zasshi	case report	1	ECS	70	F	1. hypokalemia 2. hypertension 3. hyperglycemia 4. metabolic alkalosis	(+)	1. chemotherapy	11 months	1. progressive disease	
12(28)	Sato	1997	Nihon Ronen Igakkai Zasshi	case report	1	ECS	72	M	1. hypokalemia 2. hypertension 4. metabolic alkalosis 5. edema	(+)	1. chemotherapy 2. radiotherapy 5. mitotane	26 months	5. respiratory failure	
13(29)	Cabezas	1998	Neth J Med	case report	1	ECS	56	M	1. hypokalemia 4. metabolic alkalosis 9. polyuria	(+)	1. chemotherapy 6. octreotide	9 months	1. meningitis carcinomatosa	
14(31)	Bodvarsson	2001	Cancer	case report	1✖	ECS	25	F	5. edema 6. weakness		1. chemotherapy 3. nephrectomy of the transplanted kidney	/	/	18 months
15(30)	Dubé	2001	Ann Fr Anesth Reanim	case report	1	ECS	58	M	1. hypokalemia 4. metabolic alkalosis 5. edema 7. dyspnea		/	1 week	5. respiratory deterioration	
16(68)	Sakuraba	2003	Jpn J Thorac Cardiovasc Surg	case series	1	ECS	44	F	1. hypokalemia 6. fatigue	(+)	3. surgery (anterior lobe of pituitary, adrenal gland, right middle lobectomy)	117 months	/	
17(32)	Agha	2005	Pituitary	case report	1	ECS	49	M	1. hypokalemia 2. hypertension 5. edema 6. weakness 7. dyspnea 9. polyuria	(+)	1. chemotherapy 4. ketoconazole	9 months	/	
18(73)	Ilias	2005	J Clin Endocrinol Metab	retrospective	3	ECS	/	2/1	1. hypokalemia 2. hypertension 3. diabetes 5. edema 6. muscle weakness		1. chemotherapy 3. bilateral adrenalectomy (2/3) 7. with/without endocrine therapy	/	/	1–48 months
19(34)	Hadem	2007	Z Gastroenterol	case report	1	ECS	68	F	1. hypokalemia 2. hypertension 3. hyperglycemia 4. metabolic alkalosis 6. muscle weakness 7. dyspnea		1. chemotherapy 4. ketoconazole	7 weeks	1. intracranial tumor spread 2. septic complications 3. endocrine and electrolyte disturbances 5. pneumonia	
20(35)	Lee	2007	Endocrinol Metab	case report	1	ECS	73	F	1. hypokalemia 2. hypertension 3. hyperglycemia 4. metabolic alkalosis 5. edema 6. weakness	(-)	1. chemotherapy 2. radiotherapy	10 days	5. neutropenic pneumonia	
21(36)	Muessig	2007	Internist	case report	1	ECS	68	F	1. hypokalemia 2. hypertension 3. hyperglycemia 6. weakness 7. dyspnea	(+)	1. chemotherapy	/	/	3 courses therapy
22(37)	Servonnet	2007	Ann Biol Clin	case report	1	ECS	41	F	1. hypokalemia 2. hypertension 3. hyperglycemia 4. metabolic alkalosis 5. edema 7. dyspnea		1. chemotherapy 5. metopirone	/	/	
23(33)	Tanaka	2007	Nihon Kokyuki Gakkai Zasshi,	case report	1	ECS	54	F	9. polyuria		1. chemotherapy 2. radiotherapy	3 months	2. multiple intracystic infections of bilateral upper lobe 6. general deterioration	
24(39)	Fernández-Rodríguez	2008	Arq Bras Endocrinol Metabol	case report	1	ECS	59	M	1. hypokalemia 2. hypertension 4. metabolic alkalosis 5. edema	(-)	1. chemotherapy 4. ketoconazole	<1 month	2. septic shock	
25(38)	Guabello	2008	Am J Clin Oncol	case report	1	ECS	71	M	1. hypokalemia 2. hypertension 3. hyperglycemia 5. edema		1. chemotherapy	1 month	2. blood infection, septic shock 3. hypercortisolism 6. nephrosis	
26(40)	Vadlamudi	2008	South Med J	case report	1•	ECS^▴^	76	M	1. hypokalemia 2. hypertension 4. metabolic alkalosis 6. weakness 7. dyspnea		/	several days	2. hyperinfection 3. hypercortisolism 4. cardiac arrest	
27(41)	Bindi	2009	Recenti Prog med	case report	1	ECS	64	M	1. hypokalemia 8. hypothyroidism		1. chemotherapy	/	/	
28(42)	Martínez-Valles	2009	Cases J	case report	1	ECS	54	M	1. hypokalemia 2. hypertension 4. metabolic alkalosis 5. edema 6. fatigue 9. polyuria		4. ketoconazole	a few days	2. sepsis due to a right leg cellulitis 5. respiratory failure 6. bilateral pleural effusions	
29(43)	Cicin	2010	Trak Univ Tip Fak Derg	case report	1	ECS	37	M	1. hypokalemia 4. alkalosis 5. edema 6. weakness		1. chemotherapy 4. ketoconazole	11 days	2. infection 5. pneumonia	
30(74)	Doi	2010	Endocr J	retrospective	2	ECS	58	M	/		1. chemotherapy 2. radiotherapy	7 months	1. cancer	
						ECS	69	M	1. hypokalemia 2. hypertension 3. diabetes 6. weakness		1. chemotherapy 2. radiotherapy 5. mitotane, metyrapone	6 months	1. cancer	
31(11)	Ejaz	2011	Cancer	retrospective	9	ECS	/	/	1. hypokalemia 2. hypertension 3. hyperglycemia 4. alkalosis	(+)	3. surgery (bilateral adrenalectomy, resection of primary tumors, combined bilateral adrenalectomy along with primary tumor resection) 4. ketoconazole 5. metyrapone	/	2. infections 3. hyperglycemia 4. venous thromboembolism	
32(44)	Suyama	2011	Intern Med	case report	1	ECS	53	M	1. hypokalemia 2. hypertension 3. hyperglycemia 7. dyspnea		1. chemotherapy 5. mitotane	5 months	1. cancer progress	
33(45)	Stempel	2013	BMJ Case Rep	case report	1	ECS^▴^	79	F	2. hypertension 4. metabolic alkalosis 5. edema 6.lethargy 7. dyspnea		5. metyrapone	1 week	4. lateral ischaemia 5. respiratory failure	
34(46)	Akinosoglou	2014	Ann Clin Biochem	case report	1	ECS	59	M	1. hypokalemia 5. edema		1. chemotherapy 4. ketoconazole	/	/	
35(6)	Nagy-Mignotte	2014	J Thorac Oncol	retrospective	23	CS	62 (29-84)	16/7	1. hypokalemia (95.6%) 2. hypertension (56.5%) 3. hyperglycemia (95.6%) 4. metabolic alkalosis (69.6%) 5. edema (52.4%) 6. myopathy (55%)		1. chemotherapy 2. radiotherapy 3. surgery	6.6 months (95% confidence interval, 3.2–11.4)	1. cancer (81.8%) 2. infection (45.5%) 4. cardiac (9.1%)	
36(48)	Nandagopal	2014	Am J Ther	case report	1	ECS	57	F	1. hypokalemia 2. hypertension 4. metabolic alkalosis 5. edema 6. weakness, fatigue		1. chemotherapy 2. radiotherapy	/	/	
37(47)	Vega	2014	Rev Clin Esp(Barc)	case report	1	ECS	66	M	1. hypokalemia 2. hypertension 3. hyperglycemia 4. metabolic alkalosis 5. edema 7. dyspnea		1. chemotherapy 4. ketoconazole	1 month	2. escherichia coli septicemia secondary to acute perforated diverticulitis	
38(49)	Cekerevac	2015	Acta Clin Croat	case report	1	ECS^▴^	63	M	1. hypokalemia 3. hyperglycemia 6. exhaustion 7. shortness of breath		/	2 weeks	4. heart failure 5. respiratory failture	
39(76)	Ghazi	2015	Endokrynol Pol	prospective	4	ECS	61.5 (40-65)	2/2	1. hypokalemia (50%) 3. hyperglycemia (50%) 4. metabolic alkalosis (25%) 6. muscle weakness (100%) 9. polyuria (25%)		3. postero-lateral thoracotomy (2/4) 4.ketoconazole	1.5 months (1-3)	2. sepsis 4. intractable tachyarrhythmias, heart failure (only one patient mentioned)	
40(50)	Jeong	2015	Tuberc Respir Dis (Seoul)	case report	1	ECS	69	M	1. hypokalemia 2. hypertension 4. metabolic alkalosis 6. weakness	(+)	1. chemotherapy 4. ketoconazole	15 months	/	
41(52)	Aoki	2016	Intern Med	case report	1	ECS	35	M	1. hypokalemia 2. hypertension 6. muscle weakness 9. polyuria	(+)	1. chemotherapy	4 courses therapy	6. suffocation due to a retropharyngeal abscess	
42(51)	Kaya	2016	J Clin Diagn Res	case report	1	ECS	70	M	1. hypokalaemia 2. hypertension 4. metabolic alkalosis 6. weakness	(+)	3. surgery for intestinal perforation 4. ketoconazole	12 days	6. general deterioration	
43(53)	Ohara	2016	Intern Med	case report	1	ECS	64	M	1. hypokalemia 2. hypertension 3. hyperglycemia 4. metabolic alkalosis 5. edema 6. weakness 7. dyspnea	(+)	5. metyrapone	1 month	5. idiopathic pulmonary fibrosis, respiratory failure	
44(54)	Hine	2017	J Emerg Med	case report	1	ECS	62	M	1. hypokalemia 2. hypertension 3. diabetes 5. edema 6. weakness	(+)	1. chemotherapy 2. radiotherapy	/	/	
45(56)	Wilkins	2017	Clin Schizophr Relat Psychoses	case report	1	ECS	56	M	2. hypertension 3. hyperglycemia		1. chemotherapy 2. radiotherapy 4. ketoconazole 5. metyrapone	/	/	4 courses therapy
46(55)	Zhang	2017	Thorac Cancer	case report	1	ECS	74	M	1. hypokalemia 3. hyperglycemia 4. metabolic alkalosis 5. edema 6. muscle weakness		1. chemotherapy	3 months	6. liver failure	
47(66)	Deldycke	2018	Acta Clin Belg	case series	1	ECS	71	F	1. hypokalemia 3. hyperglycemia, diabetes 5. peripheral edema 6. muscle weakness		1. chemotherapy 6. somatostatin analogue	/	/	
48(57)	Ferreira	2018	BMJ Case Rep	case report	1	ECS^▴^	42	M	1. hypokalemia 2. hypertension 4. metabolic alkalosis	(-)	1. chemotherapy 2. radiotherapy 5. metyrapone	12 months	4.5. cardiopulmonary arrest 6. clinical deterioration persisted with hypotension and prostration	
49(58)	Foray	2018	Respir Med Case Rep.	case report	1	ECS^▴^	66	F	1. hypokalemia 3. diabetes mellitus 4. metabolic alkalosis 5. edema 6. weakness 8. hypothyroidism		/	12 days	6. comorbid	
50(69)	Richa	2018	Endocrinol Diabetes Metab Case Rep	case series	2	ECS	46	F	1. hypokalemia 2. hypertension 4. metabolic alkalosis 6. lethargy, fatigue 8. hyperthyroidism		1. chemotherapy 2. radiotherapy 4. ketoconazole	12 months	/	
						ECS	51	M	1. hypokalemia 2. hypertension 3. diabetes mellitus 4. metabolic alkalosis 5. edema 6. lethargy, fatigue 7. dyspnea		1. chemotherapy 4. ketoconazole	several months	/	
51(59)	Kamijo	2019	Intern Med	case report	1	ECS	72	M	1. hypokalemia 3. hyperglycemia 6. fatigue	(+)	/	11 days	6. liver failure, hepatic and renal dysfunction	
52(70)	Zhou	2019	World J Clin Cases	case series	2	ECS	54	F	1. hypokalemia 2. hypertension 3. hyperglycemia 6. weakness 8. hypothyroidism		1. chemotherapy	/	/	2 years
						ECS	50	M	1. hypokalemia 2. hypertension 3. diabetes mellitus 5. edema 8. hypothyroidism 9. diuresis		1. chemotherapy 2. radiotherapy	/	/	4 courses therapy
53(63)	Gerhardt	2020	Dtsch Med Wochenschr	case report	1	ECS	58	M	1. hypokalemia 2. hypertension 4. metabolic alkalosis 5. edema	(+)	1. chemotherapy 4. ketoconazole	/	/	
54(61)	Kosuda	2020	Intern Med	case report	1	ECS✦	70	F	1. hypokalemia 5. edema 6. fatigue		1. chemotherapy 2. radiotherapy	40 months	1. cancer progression	
55(63)	Cabral	2020	Eur J Case Rep Intern Med	case report	1	CS	49	M	1. hypokalemia 2. hypertension 4. metabolic alkalosis 5. edema 6. asthenia 7. dyspnea 9. polyuria		5. etomidate, metyrapone	50 days	2. infection 5. pneumonia, respiratory failure	
56(60)	Pingle	2020	ESC Heart Fail	case report	1	ECS	64	M	1. hypokalemia 2. hypertension 3. hyperglycemia, diabetes mellitus 5. edema		1. chemotherapy 5. metyrapone	/	/	5 courses therapy
57(75)	Lopez-Montoya	2021	Arch Endocrinol Metab	retrospective	3	ECS	68(50-72)	2/1	1. hypokalemia 2. hypertension 3. diabetes mellitus 6. proximal myopathy		1. chemotherapy (2/3) 2. radiotherapy (1/3) 3. bilateral adrenalectomy (1/3) 4. ketoconazole (2/3)	2 months (22 days-3 months)	1. disease progression 2. febrile neutropenia, septic shock, sepsis 5. respiratory failure	
58(65)	Qiang	2021	BMC Endocr Disord	case report	1▪	ECS	64	M	1. hypokalemia 3. hyperglycemia 4. metabolic alkalosis 5. edema 6. weakness	(+)	1. chemotherapy 7. mifepristone	<1 month	6. dyscrasia	
59(64)	Senarathne	2021	BMJ Case Rep	case report	1	ECS	56	M	1. hypokalemia 3. hyperglycemia 5. edema 6. weakness 7. dyspnea	(+)	1. chemotherapy 4. ketoconazole	3 weeks	5. neutropenic pneumonia	
60(72)	Piasecka	2022	Front Med (Lausanne)	case series	1	ECS^▴^	81	M	1. hypokalemia 2. hypertension 5. edema 6. muscle weakness 7. dyspnea		/	1 week	6. condition deteriorated	
61(71)	Rosales-Castillo	2022	Hipertens Riesgo Vasc	case series	2	ECS	59	M	1. hypokalemia 2. hypertension 3. hyperglycemia 4. metabolic alkalosis 9. polyuria		1. chemotherapy	1 courses therapy	6. massive hematemesis	
						ECS	47	M	1. hypokalemia 2. hypertension 3. hyperglycemia 4. metabolic alkalosis 5. edema 6. weakness		4. ketoconazole	2 months	3. metabolic alterations	

1.▴suspected; 2.★CRH induced; 3.✦combined with SIADH; 4.✖donor-derived SCLC; 5.♦;mixed with NSCLC; 6▪mixed with large cell neuroendocrine carcinoma; 7.•mixed with adenocarcinoma and giant cell carcinoma of the lung.

N, number of patients; M, male; F, female; CS, Cushing’s syndrome; PCS, paraneoplastic Cushing’s syndrome; ECS, ectopic Cushing’s syndrome; IHC, immunohistochemical; ACTH, adrenocorticotropic hormone; CRH, corticotropin-releasing hormone; SIADH, syndrome of inappropriate antidiuretic hormone secretion; SCLC, small cell lung cancer; NSCLC, non-small cell lung cancer.

**Table 2:** The frequency of specific details recorded in all 61 reports.

Category	Specific Detail	No. of Articles	Rank
Clinical Presentation			
1	hypokalemia	59(96.72%)	1
2	hypertension	42(68.85%)	2
3	hyperglycemia or diabetes mellitus	37(60.66%)	4
4	metabolic alkalosis	37(60.66%)	4
5	edema	36(59.02%)	5
6	myopathy, weakness, fatigue, or lethargy	41(67.21%)	3
7	dyspnea	19(31.15%)	6
8	hypothyroidism or hyperthyroidism	5(8.20%)	8
9	diuresis, polyuria, polyuresis or diabetes insipidus	11(18.03%)	7
Treatment			
1	chemotherapy	50(81.97%)	1
2	radiotherapy	17(27.87%)	3
3	surgery (lung, adrenal gland or elsewhere)	9(14.75%)	5
4	ketoconazole	24(39.34%)	2
5	metyrapone, mitotane, etomidate or other steroidogenesis inhibitors	15(24.59%)	4
6	octreotide, lanreotide or other somatostatin analogues	4(6.56%)	6
7	mifepristone or other treatments for hypercortisolism	2(3.28%)	7
Cause of Death			
1	cancer	17(27.87%)	1
2	infection	17(27.87%)	1
3	hormone issues	7(11.48%)	5
4	cardiovascular complications	8(13.11%)	4
5	respiratory complications	16(26.23%)	2
6	other causes (included general condition deteriorated)	15(24.59%)	3

The percentage was calculated as the proportion of the articles with records of corresponding details out of the all 61 articles.

All 157 patients were diagnosed with SCLC through histological methods. The one reported by Bodvarsson et al. was a patient with donor-derived SCLC (31). One patient (1/10) reported by Winquist et al. was mixed SCLC with non-small cell lung cancer (NSCLC) (10). The one reported by Qiang et al. was mixed SCLC with large cell neuroendocrine carcinoma (65). The one reported by Vadlamudi et al. had combined SCLC, lung adenocarcinoma, and giant cell carcinoma of the lung (40).

For differential diagnosis of CS, 132 SCLC patients (84.1% of all 157 patients) in 58 reports (95.1% of all 61 reports) were diagnosed with ECS. In 9 (15.5%) reports, 40 (30.3%) patients were diagnosed without strict evidence from combining imaging examinations with laboratory tests or reported with no mention of specific procedures for the diagnosis of ectopic hormone secretion (8, 10, 22, 40, 45, 49, 57, 58, 72). The patient reported by Cabral et al. (63) and the 23 patients in the report by Nagy-Mignotte et al. (6) were merely diagnosed as PCS without further investigating the hormone origin. The patient reported by Tabata et al. (24) was diagnosed as PCS and his IHC result was negative for ACTH, while some laboratory tests revealed the opposite. In addition, the patient reported by Kosuda et al. was complicated not only by ECS but also by the syndrome of inappropriate antidiuretic hormone secretion (61). Moreover, IHC staining for ACTH was performed in 30 (19.1%) patients, 29 had ECS and 1 had PCS, across 22 (36.1%) reports. Out of these reports, four ECS patients (25, 35, 39, 57) and one PCS patient (24) showed negative results. It is noteworthy that the case reported by Auchus et al. stained negative for ACTH, but positive for corticotropin-releasing hormone (CRH), which confirmed that the patient’s ECS was caused by ectopic CRH secretion rather than ACTH (25).

Regarding clinical presentation, hypokalemia was mentioned in 59 (96.72%) reports as the most frequently recorded clinical feature in PCS patients, followed by hypertension in 42 (68.85%) reports. For treatment, chemotherapy and ketoconazole were the first-line option used for SCLC patients with PCS, in 50 (81.97%) and 24 (39.34%) reports, respectively. As for cause of death, infection was recorded in 17 (27.87%) reports, equally to cancer. The remaining causes included respiratory complications in 16 (26.23%) reports, cardiovascular complications in 8 (13.11%), hormone issues in 7 (11.48%), and other causes (including general condition deterioration) in 15 (24.59%).

Regarding the survival of SCLC with PCS patients, five retrospective studies (6–9, 75) and one prospective study (76) indicated unfavorable results, with median survivals of less than 7 months. However, eight case reports with superior outcomes also existed, all showing a survival of 1 year or more (28, 31, 50, 57, 61, 68–70), with four patients having lived for over 2 years (28, 61, 68, 70). The longest survival was 117 months, reported by Sakuraba et al. (61).

General descriptions of specific cases exhibiting long-term survival, mixed pathological types of lung cancer, or negative results in IHC staining for ACTH, as well as a brief introduction to the latest retrospective study, are presented in **Supplementary Materials**.

## 4 Discussion

In this systematic review, we have incorporated relevant reports of SCLC with PCS available on PubMed and Web of Science and presented a comprehensive analysis. Through the review and analysis, we have not only reflected on opportunities to refine the differential diagnostic strategy for PCS, but also discovered key considerations to underpin the comprehensive treatment of SCLC with PCS patients.

### 4.1 Differential diagnosis of ECS from Cushing’s disease

Since PCS is a marker of poor prognosis for SCLC patients, it is crucial to perform early differential diagnosis of CS to evaluate the prognosis of SCLC patients. Once the CS has been identified and the ACTH non-dependent type has been ruled out, the most challenging part is distinguishing ECS from Cushing’s disease (CD), where the pituitary gland releases ACTH (12, 57, 66). In this context, IHC staining for ACTH in the tumor tissue could be an efficient diagnostic tool.

No individual imaging examination or laboratory test has been explicitly recommended to definitively differentiate between pituitary and ectopic CS (3, 66, 77). Despite the fact that high-dose dexamethasone suppression test and CRH stimulation test may individually yield inaccurate outcomes, their combination has shown a better diagnostic performance (3, 12, 77–80). Following the Pituitary Society’s guideline, in the event of negative outcomes for both tests, consideration should be given to diagnosing ECS. Conversely, if both tests yield positive results, CD should be acknowledged. If the results are mismatched, a bilateral inferior petrosal sinus sampling is necessary for a definite diagnosis (77). Furthermore, a conclusion drawn in collaboration with imaging techniques must be more convincing. The guideline recommended magnetic resonance imaging (MRI) as the preferred modality for imaging ACTH-secreting pituitary adenomas (77) despite its high rate of false negative or false positive (12, 66, 81). Moreover, emerging data have suggested that the CRH/desmopressin stimulation test in collaboration with pituitary MRI, subsequently followed by a whole-body computed tomography scan, could be a reliable alternative (77, 82, 83). However, some investigators suggested that a conclusive diagnosis of an ACTH-secreting tumor should only be made post-surgery. After surgical removal of the tumor, the resolution of hypercortisolism symptoms and the positive IHC staining for ACTH or its precursor in excised tissues could indicate an ECS diagnosis (66, 81).

IHC staining for ACTH has demonstrated a high degree of reliability for its consistency with outcomes from the combination of laboratory tests and imaging examinations within our reviewed reports. However, none of the three guidelines from the American Endocrine Society, European Society of Endocrinology, or the Pituitary Society contained any histological diagnosis-relevant contents on ECS diagnosis in SCLC patients (13, 77, 78, 84). We look forward to this technique being evaluated by a proficient multidisciplinary team in the future and the latest guidelines shedding some light on this diagnostic method. The IHC staining of a distinctive hormone (either ACTH or CRH) allows us to make an early diagnosis of ECS in conjunction with the pathological diagnosis of SCLC and to take prophylactic measures against hypercortisolism, which can exacerbate cancer-induced immunosuppression and cause severe infectious complications afterwards (6, 8, 9, 11, 64, 85).

**Figure 2:**
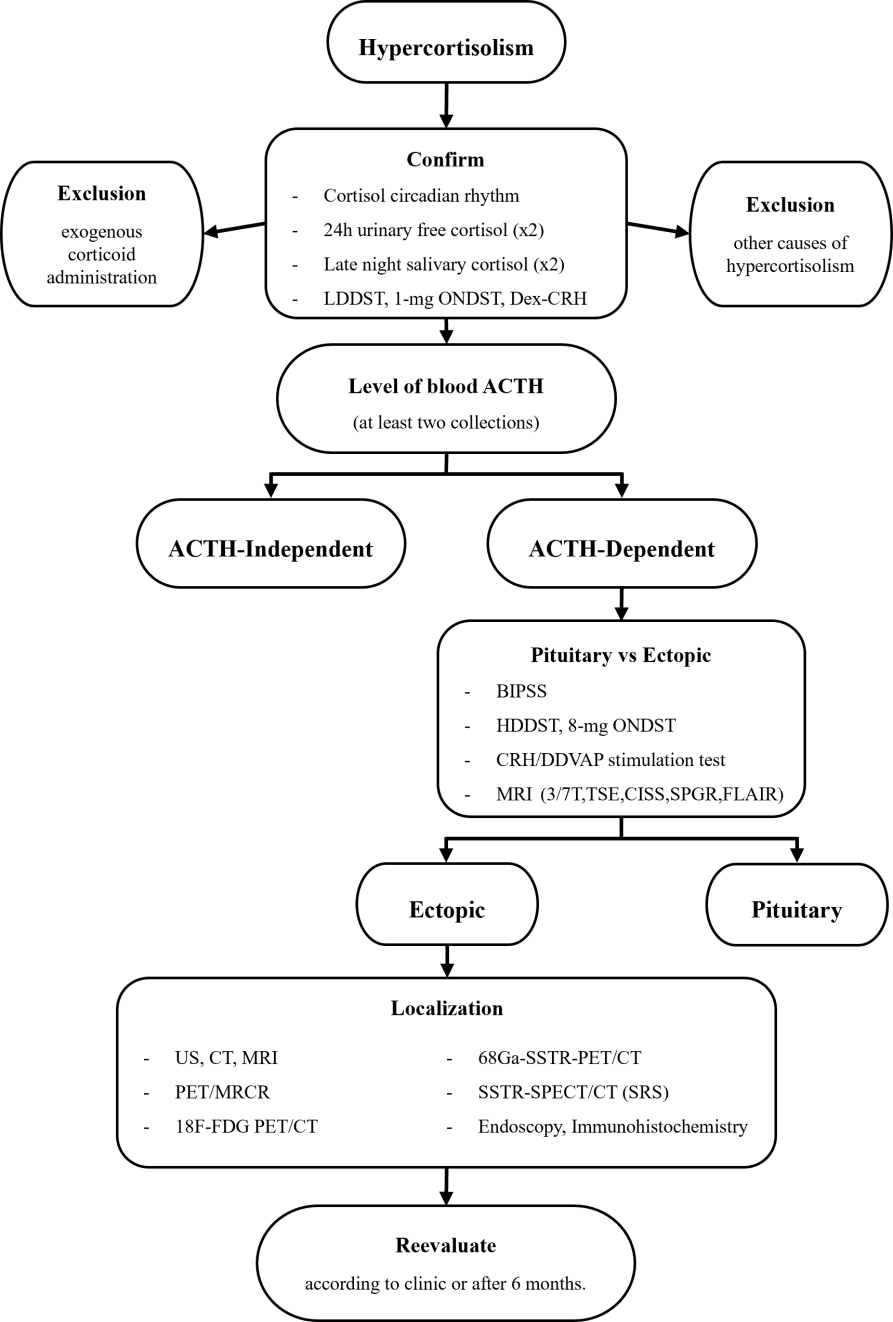
Flow diagram for multistep diagnostic procedures of ECS. Based on the figure in the review of Deldycke et al. ECS, ectopic Cushing’s syndrome; ACTH, adrenocorticotropic hormone; CRH, corticotropin-releasing hormone; DDAVP, desmopressin or 1-deamino-8-D-arginine-vasopressin; ONDST, overnight dexamethasone suppression test; LDDST, low-dose dexamethasone suppression test; HDDST, high-dose dexamethasone suppression test; Dex-CRH, combined LDDST-CRH test; BIPSS, bilateral inferior petrosal sinus sampling; US, ultrasound; CT, computed tomography; MRI, magnetic resonance imaging; TSE, T1-weighted turbo spin echo; CISS, constructive interference in the steady state; SPGR, spoiled gradient recalled; FLAIR, fluid attenuation inversion recovery; PET, positron emission tomography; SPECT, single-photon emission computed tomography; PET/MRCR, PET coregistration with volumetric MRI; 18F-FDG, 18F-fluoro-deoxy-glucose; SSTR-PET/CT, somatostatin receptor-based positron emission tomography/computed tomography (with 68Ga⁃DOTATATE/DOTATOC/DOTANOC); SSTR-SPECT/CT, somatostatin receptor-based single-photon emission computed tomography/computed tomography; SRS (octreoscan), somatostatin receptor scintigraphy (with Octreotide).

Based on the literatures reviewed and the charts appreciated (3, 11–13, 66, 78, 82, 86, 87), we improved and perfected the specific flowchart for the multistep diagnostic procedures of ECS from Deldycke et al. (66). The flowchart is displayed in **Figure 2**.

### 4.2 Therapeutic strategy for SCLC with PCS

The treatment of SCLC with PCS patients demands two primary factors. On one hand, it is vital to manage hypercortisolism and take prophylactic measures against infections. On the other hand, some therapeutic strategies have advanced in the treatment of SCLC.

On one hand, both infection and cancer ranked highest among all causes of death, with each being mentioned in 17 (27.87%) reports. Furthermore, a significant number of patients had infections recorded before respiratory complications (indicated in 16 reports). Infection facilitated by glucocorticoid-induced immunosuppression and chemotherapy-induced agranulocytosis is a significant poor prognostic factor for SCLC with PCS patients (6, 8, 9, 11, 64, 85). Therefore, management of hypercortisolism and prophylaxis against infection is particularly important throughout the entire treatment process. Many authors emphasized the importance of controlling hypercortisolism by a specific treatment before or concurrently with chemotherapy to prevent infectious complications (6, 43, 52, 64, 75, 76). Apart from radiotherapy and surgical removal, the main pharmacological treatments for PCS include steroidogenesis inhibitors (e.g., ketoconazole, metyrapone, mitotane, etomidate, and osilodrostat), glucocorticoid receptor antagonists (e.g., mifepristone), somatostatin analogs (e.g., octreotide, lanreotide, and pasireotide), and dopamine agonists (e.g., cabergoline) (3, 4, 12, 13). According to guidelines and high-quality reviews, steroidogenesis inhibitors have been the principal treatment to control hypercortisolism while somatostatin analogs and dopamine agonists are recommended to inhibit ectopic ACTH production with limited intensity (3, 4, 12, 13). Within our included reports, steroidogenesis inhibitors were used most frequent as in 39 (63.93%) reports, especially ketoconazole recorded in 24 (39.34%) reports, while somatostatin analogs and mifepristone were used little and no dopamine agonists were recorded.

On the other hand, the ultimate cause of hypercortisolism is ectopic hormone secretion by tumor tissues, meaning that the treatment for cancer is fundamental to the management of hypercortisolism and effective anti-cancer treatment could alleviate PCS symptoms. All 61 reports we examined recorded chemotherapy and radiotherapy as anti-tumor therapeutic strategies apart from surgery. In fact, there had been no substantial progress in treatment of SCLC for over 30 years until ICIs updated the treatment pattern and modestly improved its overall survival (1, 2, 14, 15). Formerly, platinum plus etoposide combination chemotherapy was the preferred regimen for both limited and extensive SCLC. Nowadays, the new standard of care in first-line setting for SCLC is immuno-chemotherapy that combines atezolizumab or durvalumab with platinum-etoposide (14, 15, 88, 89). Considering significant improvement in medical care over recent years and the introduction of immunotherapy into therapeutic strategy for SCLC, we look forward to seeing some reports, especially high-quality large-sample studies conducted by proficient teams, evaluating the efficacy and safety of some novel medical approaches, particularly the immunotherapy, for the treatment of SCLC with PCS in the future.

We retrieved all drug approval notifications for SCLC in the *Oncology (Cancer)*/*Hematologic Malignancies Approval Notifications*) section on the official website of USA Food and Drug Administration, which revealed a slow progression in treatment of SCLC compared to NSCLC, as summarized in **Supplementary Materials**.

### 4.3 Limitations

This systematic review has some limitations that need to be acknowledged in order to contextualize the conclusions that have been drawn or will be drawn from it. Firstly, the literature available on the subject is relatively limited, and there exists a significant degree of heterogeneity among the reports included in this review. This review includes several types of studies, and there are significant differences in outlines, focuses, and details of reported management of the disease, even within the same study type. Secondly, the evidence grade of case reports or case series is low. Among the 61 included reports, a significant proportion is composed of 44 case reports and 7 case series. As a result, the complete data in **Table 2** were derived from the number of reports that had records of corresponding details, rather than from the number of patients, which makes the statistical analysis sketchy and generalized. Thirdly, the included reports stretch over a period from 1985 to 2022, during which medical care rapidly evolved, which could be the inherent limitation for systematic reviews with too wide a temporal scope. Ultimately, it is essential to acknowledge that this systematic review serves only as a preliminary exploration for the management of SCLC with PCS patients, and further validation is eagerly awaited from future high-quality studies covering significant sample sizes.

## 5 Conclusions

This systematic review indicated that the majority of PCS complications in SCLC patients were caused by ectopic hormone secretion. Furthermore, it is recommended to enhance the employment of IHC staining for distinctive hormones (ACTH or CRH) in clinical practice for early differential diagnosis of PCS. Moreover, effective management of hypercortisolism and potent safeguarding against infections could form the foundation of comprehensive treatment of SCLC with PCS patients.

## Data availability statement

The original contributions presented in the study are included in the article/**Supplementary Material**. Further inquiries can be directed to the corresponding author.

## Author contributions

YL designed the study. YL and CL collected the data. CL and XQ analyzed the data. YL prepared tables and figures. YL, CL and XQ drafted the manuscript. LY and LL supervised the study and revised the manuscript. All authors read and approved the submitted version of the manuscript.

## Glossary

18F-FDG: 18F-fluoro-deoxy-glucose
ACTH: Corticotropin, adrenocorticotropic hormone
BIPSS: Bilateral inferior petrosal sinus sampling
CD: Cushing’s disease (from pituitary)
CISS: Constructive interference in the steady state
CRH: Corticotropin-releasing hormone
CS: Cushing’s syndrome
CT: Computed tomography
DDVAP: Desmopressin, 1-deamino-8-D-arginine-vasopressin
Dex-CRH: Combined LDDST-CRH test
EC: Etoposide plus carboplatin
ECS: Ectopic Cushing’s syndrome
EP: Etoposide plus cisplatin
FLAIR: Fluid attenuation inversion recovery
HDDST: High-dose dexamethasone suppression test
ICI: Immune checkpoint inhibitor
IHC staining: Immunohistochemical staining
JBI: Joanna Briggs Institute
LDDST: Low-dose dexamethasone suppression test
MRI: Magnetic resonance imaging
NSCLC: Non-small cell lung cancer
ONDST: Overnight dexamethasone suppression test
PCS: Paraneoplastic Cushing’s syndrome
PET: Positron emission tomography
PET/MRCR: PET coregistration with volumetric MRI
SCLC: Small cell lung cancer
SIADH: Syndrome of inappropriate antidiuretic hormone secretion
SPECT: Single-photon emission computed tomography
SPGR: Spoiled gradient recalled
SRS (octreoscan): Somatostatin receptor scintigraphy (with Octreotide)
SSTR-PET/CT: Somatostain receptor-based positron emission tomography/computed tomography (with 68Ga⁃DOTATATE/DOTATOC/DOTANOC)
SSTR-SPECT/CT: Somatostain receptor-based single-photon emission computed tomography/computed tomography
TSE: T1-weighted turbo spin echo
US: Ultrasound

## References

[1] Wang Q Gümüş ZH Colarossi C Memeo L Wang X Kong CY . SCLC: epidemiology, risk factors, genetic susceptibility, molecular pathology, screening, and early detection. J Thorac Oncol (2022) 18(1):31–46. doi: 10.1016/j.jtho.2022.10.002 PMC10797993 36243387

[2] Rudin CM Brambilla E Faivre-Finn C Sage J . Small-cell lung cancer. Nat Rev Dis Primers (2021) 7:3. doi: 10.1038/s41572-020-00235-0 33446664 PMC8177722

[3] Lacroix A Feelders RA Stratakis CA Nieman LK . Cushing's syndrome. Lancet (2015) 386:913–27. doi: 10.1016/S0140-6736(14)61375-1 26004339

[4] Alexandraki KI Grossman AB . The ectopic ACTH syndrome. Rev Endocr Metab Disord (2010) 11:117–26. doi: 10.1007/s11154-010-9139-z 20544290

[5] Valassi E Franz H Brue T Feelders RA Netea-Maier R Tsagarakis S . Diagnostic tests for Cushing's syndrome differ from published guidelines: data from ERCUSYN. Eur J Endocrinol (2017) 176:613–24. doi: 10.1530/EJE-16-0967 28377460

[6] Nagy-Mignotte H Shestaeva O Vignoud L Guillem P Ruckly S Chabre O . Prognostic impact of paraneoplastic cushing's syndrome in small-cell lung cancer. J Thorac Oncol (2014) 9:497–505. doi: 10.1097/JTO.0000000000000116 24736072

[7] Delisle L Boyer MJ Warr D Killinger D Payne D Yeoh JL . Ectopic corticotropin syndrome and small-cell carcinoma of the lung. Clinical features, outcome, and complications. Arch Intern Med (1993) 153:746–52. doi: 10.1001/archinte.1993.00410060054009 8383484

[8] Shepherd FA Laskey J Evans WK Goss PE Johansen E Khamsi F . Cushing's syndrome associated with ectopic corticotropin production and small-cell lung cancer. J Clin Oncol (1992) 10:21. doi: 10.1200/JCO.1992.10.1.21 1309381

[9] Dimopoulos MA Fernandez JF Samaan NA Holoye PY Vassilopoulou-Sellin R . Paraneoplastic Cushing's syndrome as an adverse prognostic factor in patients who die early with small cell lung cancer. Cancer-Am Cancer Soc (1992) 69:66–71. doi: 10.1002/1097-0142(19920101)69:1<66::aid-cncr2820690113>3.0.co;2-2 1309310

[10] Winquist EW Laskey J Crump M Khamsi F Shepherd FA . Ketoconazole in the management of paraneoplastic Cushing's syndrome secondary to ectopic adrenocorticotropin production. J Clin Oncol (1995) 13:157–64. doi: 10.1200/JCO.1995.13.1.157 7799015

[11] Ejaz S Vassilopoulou-Sellin R Busaidy NL Hu MI Waguespack SG Jimenez C . Cushing syndrome secondary to ectopic adrenocorticotropic hormone secretion. Cancer-Am Cancer Soc (2011) 117:4381–89. doi: 10.1002/cncr.26029 PMC3134535 21412758

[12] Hayes AR Grossman AB . The ectopic adrenocorticotropic hormone syndrome. Endocrin Metab Clin (2018) 47:409–25. doi: 10.1016/j.ecl.2018.01.005 29754641

[13] Nieman LK Biller BMK Findling JW Murad MH Newell-Price J Savage MO . Treatment of cushing's syndrome: an endocrine society clinical practice guideline. J Clin Endocrinol Metab (2015) 100:2807–31. doi: 10.1210/jc.2015-1818 PMC4525003 26222757

[14] Barrows ED Blackburn MJ Liu SV . Evolving role of immunotherapy in small cell lung cancer. Semin Cancer Biol (2022) 86:868–74. doi: 10.1016/j.semcancer.2022.02.021 35192928

[15] Remon J Aldea M Besse B Planchard D Reck M Giaccone G . Small cell lung cancer: a slightly less orphan disease after immunotherapy. Ann Oncol (2021) 32:698–709. doi: 10.1016/j.annonc.2021.02.025 33737119

[16] Dingemans AC Früh M Ardizzoni A Besse B Faivre-Finn C Hendriks LE . Small-cell lung cancer: ESMO Clinical Practice Guidelines for diagnosis, treatment and follow-up(☆). Ann Oncol (2021) 32:839–53. doi: 10.1016/j.annonc.2021.03.207 PMC9464246 33864941

[17] Atkinson M Lansdown AJ . Endocrine immune-related adverse events: Adrenal, parathyroid, diabetes insipidus, and lipoatrophy. Best Pract Res Clin Endocrinol Metab (2022) 36:101635. doi: 10.1016/j.beem.2022.101635 35382989

[18] Lupu J Pages C Laly P Delyon J Laloi M Petit A . Transient pituitary ACTH-dependent Cushing syndrome caused by an immune checkpoint inhibitor combination. Melanoma Res (2017) 27:649–52. doi: 10.1097/CMR.0000000000000405 29036015

[19] Paepegaey AC Dot JM Beauvy J Juttet P Le Berre JP . Pembrolizumab-induced cyclic ACTH-dependent Cushing's syndrome treated by a block-and-replace approach with osilodrostat. Ann Endocrinol (Paris) (2022) 83:73–5. doi: 10.1016/j.ando.2021.11.007 34871599

[20] Tan MH Iyengar R Mizokami-Stout K Yentz S MacEachern MP Shen LY . Spectrum of immune checkpoint inhibitors-induced endocrinopathies in cancer patients: a scoping review of case reports. Clin Diabetes Endocrinol (2019) 5:1. doi: 10.1186/s40842-018-0073-4 30693099 PMC6343255

[21] Page MJ McKenzie JE Bossuyt PM Boutron I Hoffmann TC Mulrow CD . The PRISMA 2020 statement: An updated guideline for reporting systematic reviews. PloS Med (2021) 18:e1003583. doi: 10.1371/journal.pmed.1003583 33780438 PMC8007028

[22] Shepherd FA . Ketoconazole. Use in the treatment of ectopic adrenocorticotropic hormone production and Cushing's syndrome in small-cell lung cancer. Arch Internal Med (1985) 145:863–64. doi: 10.1001/archinte.145.5.863 2986567

[23] Hoffman DM Brigham B . The use of ketoconazole in ectopic adrenocorticotropic hormone syndrome. Cancer-Am Cancer Soc (1991) 67:1447–49. doi: 10.1002/1097-0142(19910301)67:5<1447::AID-CNCR2820670531>3.0.CO;2-I 1846777

[24] Tabata M Ohnoshi T Ueoka H Kiura K Segawa Y Shibayama T . [A case of small cell lung cancer associated with diabetes insipidus and Cushing's syndrome]. Nihon Kyobu Shikkan Gakkai Zasshi (1993) 31:235–39. 8390589

[25] Auchus RJ Mastorakos G Friedman TC Chrousos GP . Corticotropin-releasing hormone production by a small cell carcinoma in a patient with ACTH-dependent Cushing’s syndrome. J Endocrinol Invest (1994) 17:447–52. doi: 10.1007/BF03347737 7930390

[26] Huang TP Wang PW Liu RT Tung SC Jean WY Lu YC . Ectopic ACTH syndrome with nocardiosis–a case report. Changgeng Yi Xue Za Zhi (1994) 17:371–77. 7850654

[27] Takano K Takayama K Nakano H Hagimoto N Nakanishi Y Hara N . Small cell lung cancer associated with ectopic ACTH syndrome. Nihon Kyōbu Shikkan Gakkai zasshi (1996) 34:220–25. 8622281

[28] Sato S Yokoyama A Ohtsuka T Nomoto T Abe M Kohno N . Cushing's syndrome due to small cell lung cancer with ectopic production of adrenocorticotropic and parathyroid hormone. Nippon Ronen Igakkai Zasshi. Japanese J Geriatrics (1997) 34:215–20. doi: 10.3143/geriatrics.34.215 9155197

[29] Castro Cabezas M Vrinten DH Burgers JA Croughs RJ . Central diabetes insipidus and Cushing's syndrome due to ectopic ACTH production by disseminated small cell lung cancer: a case report. Neth J Med (1998) 53:32. doi: 10.1016/S0300-2977(98)00051-5 9718940

[30] Dube L Daenen S Kouatchet A Soltner C Alquier P . [Severe metabolic alkalosis following hypokalemia from a paraneoplastic Cushing syndrome]. Ann Fr Anesth Reanim (2001) 20:860–64. doi: 10.1016/s0750-7658(01)00518-4 11803847

[31] Bodvarsson S Burlingham W Kusaka S Hafez GR Becker BN Pintar T . Donor-derived small cell lung carcinoma in a kidney transplant recipient. Cancer-Am Cancer Soc (2001) 92:2429–34. doi: 10.1002/1097-0142(20011101)92:9<2429::aid-cncr1592>3.0.co;2-g 11745300

[32] Agha A Brennan S Moore KB Grogan L Thompson CJ . Small-cell lung cancer presenting as diabetes insipidus and Cushing's syndrome. Pituitary (2005) 8:105–07. doi: 10.1007/s11102-005-3308-1 16195775

[33] Tanaka H Kobayashi A Bando M Hosono T Tsujita A Yamasawa H . [Case of small cell lung cancer complicated with diabetes insipidus and Cushing syndrome due to ectopic adrenocorticotropic hormone secretion]. Nihon Kokyuki Gakkai Zasshi (2007) 45:793–98. 18018629

[34] Hadem J Cornberg M Länger F Schedel I Kirchhoff T Niedermeyer J . Making sense of muscle fatigue and liver lesions. Z für Gastroenterologie (2007) 45:609. doi: 10.1055/s-2006-927284 17620225

[35] Yang HJ Sung HJ Kim JE Lee HJ Park JM Park CK . A case of ectopic ACTH syndrome associated with small cell lung cancer presented with hypokalemia. Endocrinology and Metabolism (2007) 22:359–64. doi: 10.3803/jkes.2007.22.5.359

[36] Müssig K Maser-Gluth C Hartmann M Wehrmann M Horger M Kanz L . 68-year-old female patient with dyspnea and hypokalemic hypertension. Der Internist (2007) 48:1145–50. doi: 10.1007/s00108-007-1930-x 17726596

[37] Servonnet A Delacour H Roux X Dehan C Gardet V Morand C . Ectopic ACTH syndrome and severe hypokalaemia. Annales biologie clinique (Paris) (2007) 65:425. doi: 10.1684/abc.2007.0142 17627926

[38] Guabello G Brunetti L Palladini G Musumeci S Lovati E Perfetti V . Paraneoplastic cushing’s syndrome and nephrotic syndrome in a patient with disseminated small cell lung cancer. Am J Clin Oncol (2008) 31:102–03. doi: 10.1097/01.coc.0000203741.06225.8a 18376237

[39] Fernandez-Rodriguez E Villar-Taibo R Pinal-Osorio I Cabezas-Agricola JM Anido-Herranz U Prieto A . Severe hypertension and hypokalemia as first clinical manifestations in ectopic Cushing's syndrome. Arq Bras Endocrinol Metabol (2008) 52:1066–70. doi: 10.1590/s0004-27302008000600019 18820819

[40] Vadlamudi RS Van Dort M Barklow T Byrd RJ Moorman JP . Strongyloides hyperinfection syndrome complicating (ectopic) Cushing syndrome. South Med J (2008) 101:750–52. doi: 10.1097/SMJ.0b013e31817a836e 18580721

[41] Bindi M Moruzzo D Pinelli M Rosada J Castiglioni M . [Hypokalemia from ectopic ACTH secretion and hypothiroidism in patient affected by small cell lung cancer]. Recenti Prog Med (2009) 100:137–39. 19475841

[42] Martínez-Valles MA Palafox-Cazarez A Paredes-Avina JA . Severe hypokalemia, metabolic alkalosis and hypertension in a 54 year old male with ectopic ACTH syndrome: a case report. cases J (2009) 2:6174. doi: 10.4076/1757-1626-2-6174 19829770 PMC2740289

[43] Cicin I Uzunoglu S Ermantas N Usta U Temizoz O Karagol H . A destroyer immunologic cause in small cell lung carcinoma: ecthopic cushing's syndrome. Med J Trakya Univ (2010) 27:312–14. doi: 10.5174/tutfd.2008.00718.1

[44] Suyama K Naito Y Yoh K Niho S Goto K Ohmatsu H . Development of Cushing's syndrome during effective chemotherapy for small cell lung cancer. Intern Med (2011) 50:335–38. doi: 10.2169/internalmedicine.50.4127 21325767

[45] von Stempel C Perks C Corcoran J Grayez J . Cardio-respiratory failure secondary to ectopic Cushing's syndrome as the index presentation of small-cell lung cancer. Case Rep (2013) 2013):r2013009974. doi: 10.1136/bcr-2013-009974 PMC3762178 23946525

[46] Akinosoglou K Siagris D Geropoulou E Kosmopoulou O Velissaris D Kyriazopoulou V . Hyperamylasaemia and dual paraneoplastic syndromes in small cell lung cancer. Ann Clin Biochem: Int J Lab Med (2014) 51:101–05. doi: 10.1177/0004563213500658 24048720

[47] Pérez Vega C . Panhipopituitarismo reversible en un paciente con síndrome de Cushing por secreción ectópica de hormona adrenocorticotropa secundaria a un carcinoma microcítico de pulmón. Rev Clínica Española (2014) 214:e5–08. doi: 10.1016/j.rce.2013.10.007 24365756

[48] Nandagopal L Arias C Pillai U Osman-Malik Y . Ectopic ACTH and cisplatin toxicity—A diagnostic dilemma. Am J Ther (2014) 21:e154–56. doi: 10.1097/MJT.0b013e3182691b03 23591027

[49] Cekerevac I Petrović M Novković L Bubanja D Bubanja I Djokić B . ECTOPIC ACTH SECRETION WITH CONCOMITANT HYPERAMYLASEMIA IN A PATIENT WITH SMALL CELL LUNG CARCINOMA: CASE REPORT. Acta clinica Croatica (Tisak) (2015) 54:536–40. 27017732

[50] Jeong C Lee J Ryu S Lee HY Shin AY Kim JS . A case of ectopic adrenocorticotropic hormone syndrome in small cell lung cancer. Tuberculosis Respir Dis (2015) 78:436–39. doi: 10.4046/trd.2015.78.4.436 PMC4620347 26508941

[51] Kaya T . Severe hypokalaemia, hypertension, and intestinal perforation in ectopic adrenocorticotropic hormone syndrome. J OF Clin AND Diagn Res (2016) 10(1):OD09–11. doi: 10.7860/JCDR/2016/17198.7127 PMC4740641 26894113

[52] Aoki M Fujisaka Y Tokioka S Hirai A Henmi Y Inoue Y . Small-cell lung cancer in a young adult nonsmoking patient with ectopic adrenocorticotropin (ACTH) production. Internal Med (Tokyo 1992) (2016) 55:1337–39. doi: 10.2169/internalmedicine.55.6139 27181543

[53] Ohara N Kaneko M Sato K Usuda H Tanaka J Maekawa T . Acute exacerbation of idiopathic pulmonary fibrosis following treatment for cushing's syndrome. Internal Med (2016) 55:389–94. doi: 10.2169/internalmedicine.55.5566 26875965

[54] Hine J Schwell A Kairys N . An unlikely cause of hypokalemia. J Emergency Med (2017) 52:e187–91. doi: 10.1016/j.jemermed.2016.12.011 28139270

[55] Zhang HY Zhao J . Ectopic Cushing syndrome in small cell lung cancer: A case report and literature review. Thorac Cancer (2017) 8:114–17. doi: 10.1111/1759-7714.12403 PMC5334324 28102935

[56] Wilkins CM Johnson VL Fargason RE Birur B . Psychosis as a sequelae of paraneoplastic syndrome in Small- Cell Lung Carcinoma: A psycho-neuroendocrine interface. Clin Schizophr Related Psychoses (2017). doi: 10.3371/CSRP.CWVJ.111717 29164929

[57] Lobo Ferreira T Nunes Da Silva T Canário D Francisca Delerue M . Hypertension and severe hypokalaemia associated with ectopic ACTH production. BMJ Case Rep (2018) 2018:bcr2017223406. doi: 10.1136/bcr-2017-223406 PMC6101307 30115708

[58] Foray N Stone T Johnson A Ali M Kulkarni S Gao J . Severe metabolic alkalosis–a diagnostic dilemma. Respir Med Case Rep (2018) 25:177–80. doi: 10.1016/j.rmcr.2018.08.019 PMC6122389 30186758

[59] Kamijo S Hasuike S Nakamura K Takaishi Y Yamada Y Ozono Y . Acute liver failure due to severe hepatic metastasis of small-cell lung cancer producing adrenocorticotropic hormone complicating ectopic cushing syndrome. Internal Med (2019) 58:2977–82. doi: 10.2169/internalmedicine.1976-18 PMC6859385 31243230

[60] Pingle SR Shah T Mosleh W Kim AS . Cushing syndrome cardiomyopathy: an unusual manifestation of small-cell lung cancer. ESC Heart Failure (2020) 7:3189–92. doi: 10.1002/ehf2.12860 PMC7524218 32573943

[61] Kosuda A Shirahata T Kudo N Uehara Y Miyawaki M Hagiwara A . Long-term survival of a patient with small cell lung cancer secreting ADH and ACTH simultaneously, following the prolonged use of amrubicin. Internal Med (2020) 59:107–12. doi: 10.2169/internalmedicine.2838-19 PMC6995698 31511478

[62] Gerhardt LMS Sabath L Müller B Capraro J Borm K . Paraneoplastisches Cushing-Syndrom als Ursache von therapierefraktärer Hypokaliämie. DMW - Deutsche Medizinische Wochenschrift (2020) 145:783–86. doi: 10.1055/a-1163-9873 32492750

[63] Lemos CS Deveza N Baptista JP Martins P . Disseminated strongyloides stercoralis infection associated with endogenous hypercortisolism - A case report. Eur J Case Rep Intern Med (2020) 7:1509. doi: 10.12890/2020_001509 PMC7350976 32665921

[64] Senarathne UD Dayanath BKTP Punchihewa R Gunasena B . Patient with respiratory distress, facial oedema and refractory hypokalaemia. BMJ Case Rep (2021) 14:e240330. doi: 10.1136/bcr-2020-240330 PMC8108648 33962921

[65] Qiang W Song S Chen T Wang Z Feng J Zhang J . A rare case of ectopic ACTH syndrome with rhabdomyolysis. BMC Endocr Disord (2021) 21:98. doi: 10.1186/s12902-021-00755-0 33971870 PMC8111963

[66] Deldycke A Haenebalcke C Taes Y . Paraneoplastic Cushing syndrome, case-series and review of the literature. Acta clinica belgica (English Ed Online) (2018) 73:298–304. doi: 10.1080/17843286.2017.1373927 28895465

[67] Rieu M Rosilio M Richard A Vannetzel J Kuhn J . Paradoxical effect of somatostatin analogues on the ectopic secretion of corticotropin in two cases of small cell lung carcinoma. Hormone Res (1993) 39:207–12. doi: 10.1159/000182737 8314205

[68] Sakuraba M Murasugi M Oyama K Adachi T Ikeda T Onuki T . Diagnosis and surgical treatment of ectopic adrenocorticotropic hormone-producing pulmonary tumors accompanied by Cushing syndrome. Japanese J Thorac Cardiovasc Surg (2003) 51:656–59. doi: 10.1007/s11748-003-0004-9 14717419

[69] Richa CG Saad KJ Halabi GH Gharios EM Nasr FL Merheb MT . Case-series of paraneoplastic Cushing syndrome in small-cell lung cancer. Endocrinol Diabetes Metab Case Rep (2018) 2018:18–0004. doi: 10.1530/EDM-18-0004 PMC5843798 29535866

[70] Zhou T Wang Y Zhao X Liu Y Wang YX Gang XK . Small cell lung cancer starting with diabetes mellitus: Two case reports and literature review. World J Clin cases (2019) 7:1213–20. doi: 10.12998/wjcc.v7.i10.1213 PMC6547319 31183355

[71] Rosales-Castillo A Bustos-Merlo A . Arterial hypertension of infrequent cause. Hipertensión y Riesgo Vasc (2022) 39:92–4. doi: 10.1016/j.hipert.2021.09.002 34656459

[72] Piasecka M Larsson M Papakokkinou E Olsson L Ragnarsson O . Is ectopic Cushing’s syndrome underdiagnosed in patients with small cell lung cancer? Front Med (2022) 9:954033. doi: 10.3389/fmed.2022.954033 PMC9468750 36111117

[73] Ilias I Torpy DJ Pacak K Mullen N Wesley RA Nieman LK . Cushing’s syndrome due to ectopic corticotropin secretion: twenty years’ Experience at the national institutes of health. J Clin Endocrinol Metab (2005) 90:4955–62. doi: 10.1210/jc.2004-2527 15914534

[74] Doi M Sugiyama T Izumiyama H Yoshimoto T Hirata Y . Clinical features and management of ectopic ACTH syndrome at a single institute in Japan. Endocr J (2010) 57:1061–69. doi: 10.1507/endocrj.K10E-265 21076235

[75] Lopez-Montoya V Gutierrez-Restrepo J Grajales JLT Aristizabal N Pantoja D Roman-Gonzalez A . Ectopic cushing syndrome in Colombia. Arch Endocrinol Metab (2020) 64(6):687–94. doi: 10.20945/2359-3997000000271 PMC10528621 34033277

[76] Ghazi AA Abbasi Dezfooli A Amirbaigloo A Daneshvar Kakhki A Mohammadi F Tirgari F . Ektopowy zespół Cushinga u pacjentów z nowotworem śródpiersia lub płuc — doniesienie z ośrodka trzeciego stopnia referencyjności w Iranie. Endokrynol Pol (2015) 66:2–09. doi: 10.5603/EP.2015.0002 25754275

[77] Fleseriu M Auchus R Bancos I Ben-Shlomo A Bertherat J Biermasz NR . Consensus on diagnosis and management of Cushing's disease: a guideline update. Lancet Diabetes Endocrinol (2021) 9:847–75. doi: 10.1016/S2213-8587(21)00235-7 PMC8743006 34687601

[78] Nieman LK Biller BMK Findling JW Newell-Price J Savage MO Stewart PM . The diagnosis of cushing's syndrome: an endocrine society clinical practice guideline. J Clin Endocrinol Metab (2008) 93:1526–40. doi: 10.1210/jc.2008-0125 PMC2386281 18334580

[79] Ejaz S Vassilopoulou-Sellin R Busaidy NL Hu MI Waguespack SG Jimenez C . Cushing syndrome secondary to ectopic adrenocorticotropic hormone secretion: the University of Texas MD Anderson Cancer Center Experience. Cancer-Am Cancer Soc (2011) 117:4381–89. doi: 10.1002/cncr.26029 PMC3134535 21412758

[80] Arnaldi G Angeli A Atkinson AB Bertagna X Cavagnini F Chrousos GP . Diagnosis and complications of Cushing's syndrome: a consensus statement. J Clin Endocrinol Metab (2003) 88:5593–602. doi: 10.1210/jc.2003-030871 14671138

[81] Witek P Witek J Zieliński G Podgajny Z Kamiński G . Ectopic Cushing's syndrome in light of modern diagnostic techniques and treatment options. Neuro Endocrinol Lett (2015) 36:201–08. 26313384

[82] Pinelli S Barbot M Scaroni C Ceccato F . Second-line tests in the diagnosis of adrenocorticotropic hormone-dependent hypercortisolism. Ann Lab Med (2021) 41:521–31. doi: 10.3343/alm.2021.41.6.521 PMC8203434 34108279

[83] Frete C Corcuff JB Kuhn E Salenave S Gaye D Young J . Non-invasive diagnostic strategy in ACTH-dependent cushing's syndrome. J Clin Endocrinol Metab (2020) 105(10):dgaa409. doi: 10.1210/clinem/dgaa409 32594169

[84] Guignat L Bertherat J . The diagnosis of Cushing's syndrome: an Endocrine Society Clinical Practice Guideline: commentary from a European perspective. Eur J Endocrinol (2010) 163:9–13. doi: 10.1530/EJE-09-0627 20375177

[85] Collichio FA Woolf PD Brower M . Management of patients with small cell carcinoma and the syndrome of ectopic corticotropin secretion. Cancer-Am Cancer Soc (1994) 73:1361–67. doi: 10.1002/1097-0142(19940301)73:5<1361::aid-cncr2820730509>3.0.co;2-j 8111702

[86] Wagner-Bartak NA Baiomy A Habra MA Mukhi SV Morani AC Korivi BR . Cushing syndrome: diagnostic workup and imaging features, with clinical and pathologic correlation. Am J roentgenol (1976) (2017) 209:19. doi: 10.2214/AJR.16.17290 28639924

[87] Isidori AM Kaltsas GA Pozza C Frajese V Newell-Price J Reznek RH . The ectopic adrenocorticotropin syndrome: clinical features, diagnosis, management, and long-term follow-up. J Clin Endocrinol Metab (2006) 91:371–77. doi: 10.1210/jc.2005-1542 16303835

[88] Wong SK Iams WT . Front line applications and future directions of immunotherapy in small-cell lung cancer. Cancers (Basel) (2021) 13(3):506. doi: 10.3390/cancers13030506 33572705 PMC7865814

[89] Esposito G Palumbo G Carillio G Manzo A Montanino A Sforza V . Immunotherapy in small cell lung cancer. Cancers (Basel) (2020) 12(9):2522. doi: 10.3390/cancers12092522 32899891 PMC7565004

