# Supplementary material for: Management of small cell lung cancer complicated with paraneoplastic Cushing’s syndrome: a systematic literature review
Source: Front Endocrinol (Lausanne). 2023 Oct 17;14:1177125. doi: 10.3389/fendo.2023.1177125 (PMC10617025; doi:10.3389/fendo.2023.1177125)
Supplement: Supplementary file 1 [file DataSheet_1.docx]

Supplementary Materials for

Management of small cell lung cancer complicated with paraneoplastic Cushing’s syndrome: a systematic literature review

Specific cases and the latest retrospective study

1.1 Specific cases

1.1.1 Cases with long survival

The longest-lived one, the patient 17 reported by Sakuraba et al. (1), was a 44-year-old woman diagnosed with alopecia, hypokalemia, and general fatigue. She was diagnosed as SCLC by transbronchial lung biopsy and identified as ECS by negative in CRH test and positive in IHC staining for ACTH. At the time of diagnosis, her SCLC was classified as T1N1M0-2A with the tumor size of 20*15*15 mm. She underwent a right middle lobectomy and node dissection, and after the surgery lived for 117 months without experiencing a recurrence of CS.

The patient 58, a 70-year-old woman reported by Kosuda et al. (2), was diagnosed as limited stage SCLC (cT2aN3M0) complicated with both ECS and SIADH. She did not receive surgery. In addition to the treatment for hyponatremia and hypokalemia, etoposide plus cisplatin (EP) chemotherapy was also initiated, along with expedited hyperfractionated radiotherapy for lung and brain. After the third cycle of chemotherapy, amrubicin were used as a second-line treatment for relapsed SCLC according to her condition. No cardiac adverse events were recorded for 2 years and 5 months after the initial administration of amrubicin. Subsequently, both blood levels of ACTH and ADH increased. The patient then received radiation for adrenal metastasis followed by amrubicin with trimethoprim-sulfamethoxazole, fluconazole, and metyrapone to prevent infection. Ultimately, she died after three years and four months after initial diagnosis, due to cancer progression.

1.1.2 Cases with mixed pathological types of lung cancer

There were four patients with heterogeneity in the diagnosis of SCLC. Patient 15, reported by Bodvarsson et al. (3), was a 25-year-old female living-related kidney transplant recipient, who was diagnosed with donor-derived SCLC of the transplanted kidney with ECS while the donor had been diagnosed with SCLC ten months after transplantation. Following six cycles of EP regime, the hypercortisolemia resolved, and the donor-derived SCLC achieved complete remission. During the last follow-up, 18 months from her diagnosis, she was asymptomatic. Patient 27, reported by Vadlamudi et al. (4), was a 76-year-old man diagnosed with Strongyloides hyperinfection syndrome complicating ECS. Although he received sustaining treatment, his health deteriorated progressively, leading to cardiac arrest and death a few days after admission. An autopsy diagnosed Strongyloides stercoralis larvae in the lung, combined with SCLC, adenocarcinoma and giant cell carcinoma of the lung. Patient 58, reported by Qiang et al. (5), was a 64-year-old man diagnosed with small-cell and large-cell neuroendocrine carcinoma by pathology and identified as ECS by high-dose dexamethasone suppression test (HDDST), bilateral inferior petrosal sinus sampling (BIPSS) and IHC staining for ACTH. The patient received mifepristone and underwent one cycle of EP regime. However, he could not tolerate further chemotherapy and succumbed to dyscrasia within a month. One 61-year-old male patient (1/10) in the report by Winquist et al. (6) was diagnosed as mixed SCLC and NSCLC. The patient received two cycles of combination chemotherapy (cyclophosphamide + doxorubicin + vincristine) with concurrent ketoconazole. However he expired before oncological reassessment could be completed.

1.1.3 Cases with negative results in IHC staining for ACTH

Four out of five patients with negative IHC staining for ACTH were diagnosed with ECS. Patient 9, reported by Auchus et al. (7), exhibited a positive result for CRH in a specific immunohistochemistry of the tumor cells from the metastatic lesion of the liver, but negative for ACTH. The patient received an EP regime for SCLC and metyrapone for ECS. However, the report did not indicate her survival or further care. Patients 21, 25 and 50, reported by Lee et al. (8), Fernández-Rodríguez et al. (9) and Ferreira et al. (10), tested negative for ACTH in IHC staining. Patient 21 received radiotherapy and oral etoposide but died on the tenth day due to leukopenia and pneumonia that developed on the seventh day. Patient 25 received etoposide and carboplatin (EC) regimen and ketoconazole. However, he developed febrile neutropenia probably due to respiratory infection after being discharged. The health deteriorated, and he died of septic shock within a month from diagnosis. Patient 50 underwent metyrapone combined with EC regime (6 cycles), topotecan (4 cycles), EC regime (2 cycles), carboplatin+paclitaxel (1 cycle), and prophylactic cranial irradiation sequentially. Unfortunately, clinical deterioration persisted with hypotension and prostration, and the patient succumbing to cardiopulmonary arrest after a year from diagnosis. These 3 patients did not receive IHC staining for CRH. As for Patient 7, reported by Tabata et al. (11), PCS diagnosis was based on the negative IHC staining for ACTH. However, when the supernatant of tumor cells obtained from culture of bone marrow puncture fluid collected during the initial consultation was analyzed, the ACTH concentration increased to 62 pg/ml compared to the control concentration of 29 pg/ml, which suggests that the ACTH was produced by tumor cells.

1.2 The latest retrospective study

The study conducted by Nagy-Mignotte and published on *Journal of Thoracic Oncology* in 2014 (12) is the most recent retrospective study on the management of SCLC with PCS patients. The study included 383 SCLC patients diagnosed between 1998 and 2012, of which 23 had PCS, 56 had other paraneoplastic syndromes, and 304 had no paraneoplastic syndrome. The results of the study showed that, compared to the other two groups, PCS patients exhibited more extensive disease (82.6% versus 67.8% and 53.3%, p = 0.005), higher prevalence of metastases (63.2% versus 15.8% and 24.1%, p ≤ 0.001), worse World Health Organization performance status (73.9% versus 57.1% and 43.7%, p = 0.006), greater incidence of weight loss (47.8% versus 33.9% and 16.4%, p ≤ 0.001), declined response and poorer sensitivity to first-line treatment (47.6% versus 74.1% and 71.1%, p = 0.04; 19% versus 38.9% and 48.6%, p = 0.01). The median survival of PCS patients was 6.6 months, as compared to 9.2 months for those with other paraneoplastic syndromes and 13.1 months for those without paraneoplastic syndrome (p ≤ 0.001). PCS was identified to be a significant prognostic marker for mortality (hazard ratio: 2.31; p ≤ 0.001). The findings suggest that PCS is a particularly severe form of paraneoplastic syndrome, characterized by extensive tumors and a diminished response to both first-line and second-line treatments.

**REFERENCES**

1. Sakuraba M, Murasugi M, Oyama K, Adachi T, Ikeda T, Onuki T. Diagnosis and surgical treatment of ectopic adrenocorticotropic hormone-producing pulmonary tumors accompanied by Cushing syndrome. *The Japanese Journal of Thoracic and Cardiovascular Surgery* (2003) **51**: 656-59. doi:10.1007/s11748-003-0004-9

2. Kosuda A, Shirahata T, Kudo N, Uehara Y, Miyawaki M, Hagiwara A, Murakami R, Shimizu K. Long-term Survival of a Patient with Small Cell Lung Cancer Secreting ADH and ACTH Simultaneously, Following the Prolonged Use of Amrubicin. *Internal Med* (2020) **59**: 107-12. doi:10.2169/internalmedicine.2838-19

3. Bodvarsson S, Burlingham W, Kusaka S, Hafez GR, Becker BN, Pintar T, Sollinger HW, Albertini MR. Donor-derived small cell lung carcinoma in a kidney transplant recipient. *Cancer-Am Cancer Soc* (2001) **92**: 2429-34. doi:10.1002/1097-0142(20011101)92:9<2429::aid-cncr1592>3.0.co;2-g

4. Vadlamudi RS, Van Dort M, Barklow T, Byrd RJ, Moorman JP. Strongyloides hyperinfection syndrome complicating (ectopic) Cushing syndrome. *South Med J* (2008) **101**: 750-52. doi:10.1097/SMJ.0b013e31817a836e

5. Qiang W, Song S, Chen T, Wang Z, Feng J, Zhang J, Guo H. A rare case of ectopic ACTH syndrome with rhabdomyolysis. *Bmc Endocr Disord* (2021) **21**. doi:10.1186/s12902-021-00755-0

6. Winquist EW, Laskey J, Crump M, Khamsi F, Shepherd FA. Ketoconazole in the management of paraneoplastic Cushing's syndrome secondary to ectopic adrenocorticotropin production. *J Clin Oncol* (1995) **13**: 157-64. doi:10.1200/JCO.1995.13.1.157

7. Auchus RJ, Mastorakos G, Friedman TC, Chrousos GP. Corticotropin-releasing hormone production by a small cell carcinoma in a patient with ACTH-dependent Cushing’s syndrome. *J Endocrinol Invest* (1994) **17**: 447-52. doi:10.1007/BF03347737

8. Lee H, 양홍준, 성혜정, 김지은, 박진민, 박찬권, 노은숙, 조재형, 고승현, 송기호 et al. A Case of Ectopic ACTH Syndrome Associated with Small Cell Lung Cancer Presented with Hypokalemia (2007) **22**: 359-64.

9. Fernandez-Rodriguez E, Villar-Taibo R, Pinal-Osorio I, Cabezas-Agricola JM, Anido-Herranz U, Prieto A, Casanueva FF, Araujo-Vilar D. Severe hypertension and hypokalemia as first clinical manifestations in ectopic Cushing's syndrome. *Arq Bras Endocrinol Metabol* (2008) **52**: 1066-70. doi:10.1590/s0004-27302008000600019

10. Lobo Ferreira T, Nunes Da Silva T, Canário D, Francisca Delerue M. Hypertension and severe hypokalaemia associated with ectopic ACTH production. *BMJ Case Reports* (2018): 2017-223406. doi:10.1136/bcr-2017-223406

11. Tabata M, Ohnoshi T, Ueoka H, Kiura K, Segawa Y, Shibayama T, Maeda T, Miyatake K, Takigawa N, Kimura I. [A case of small cell lung cancer associated with diabetes insipidus and Cushing's syndrome]. *Nihon Kyobu Shikkan Gakkai Zasshi* (1993) **31**: 235-39.

12. Nagy-Mignotte H, Shestaeva O, Vignoud L, Guillem P, Ruckly S, Chabre O, Sakhri L, Duruisseaux M, Mousseau M, Timsit JF et al. Prognostic impact of paraneoplastic cushing's syndrome in small-cell lung cancer. *J Thorac Oncol* (2014) **9**: 497-505. doi:10.1097/JTO.0000000000000116

**Supplementary Table 1.** Quality of evidence in the included 61 reports based on JBI’s critical appraisal tools.

| **Study Design** | **ID** | **First Author** | **Year** | **Journal** | **Evaluation for Evidence Reported** | | | | | | | | | | |
| --- | --- | --- | --- | --- | --- | --- | --- | --- | --- | --- | --- | --- | --- | --- | --- |
|  |  |  |  |  | **1** | **2** | **3** | **4** | **5** | **6** | **7** | **8** | **9** | **10** | **11** |
| case report | 1(1) | Shepherd | 1985 | Arch Intern Med | Y | Y | Y | Y | Y | Y | N | Y | / | / | / |
| case report | 2(2) | Hoffman | 1991 | Cancer | Y | Y | Y | Y | Y | Y | UN | Y | / | / | / |
| case report | 7(3) | Tabata | 1993 | Nihon Kyobu Shikkan Gakkai Zasshi | Y | Y | Y | Y | Y | Y | UN | UN | / | / | / |
| case report | 8(4) | Auchus | 1994 | J Endocrinol Invest | Y | Y | Y | Y | Y | Y | N | Y | / | / | / |
| case report | 9(5) | Huang | 1994 | Changgeng yi xue za zhi | Y | Y | Y | Y | Y | Y | N | UN | / | / | / |
| case report | 11(6) | Takano | 1996 | Nihon Kyobu Shikkan Gakkai Zasshi | Y | Y | Y | Y | Y | Y | N | UN | / | / | / |
| case report | 12(7) | Sato | 1997 | Nihon Ronen Igakkai Zasshi | Y | Y | Y | Y | Y | Y | UN | UN | / | / | / |
| case report | 13(8) | Cabezas | 1998 | Neth J Med | Y | Y | Y | Y | Y | Y | UN | Y | / | / | / |
| case report | 14(9) | Bodvarsson | 2001 | Cancer | Y | Y | Y | Y | Y | Y | N | UN | / | / | / |
| case report | 15(10) | Dubé | 2001 | Ann Fr Anesth Reanim | Y | Y | Y | Y | Y | Y | UN | Y | / | / | / |
| case report | 17(11) | Agha | 2005 | Pituitary | Y | Y | Y | Y | Y | Y | Y | Y | / | / | / |
| case report | 19(12) | Hadem | 2007 | Z Gastroenterol | Y | Y | Y | Y | Y | Y | UN | Y | / | / | / |
| case report | 20(13) | Lee | 2007 | Endocrinol Metab | Y | Y | Y | Y | Y | Y | UN | Y | / | / | / |
| case report | 21(14) | Muessig | 2007 | Internist | Y | Y | Y | Y | Y | Y | N | Y | / | / | / |
| case report | 22(15) | Servonnet | 2007 | Ann Biol Clin | Y | Y | Y | Y | Y | Y | N | Y | / | / | / |
| case report | 23(16) | Tanaka | 2007 | Nihon Kokyuki Gakkai Zasshi, | Y | Y | Y | Y | Y | Y | UN | Y | / | / | / |
| case report | 24(17) | Fernández-Rodríguez | 2008 | Arq Bras Endocrinol Metabol | Y | Y | Y | Y | Y | Y | UN | Y | / | / | / |
| case report | 25(18) | Guabello | 2008 | Am J Clin Oncol | Y | Y | Y | Y | Y | Y | UN | Y | / | / | / |
| case report | 26(19) | Vadlamudi | 2008 | South Med J | Y | Y | Y | Y | Y | Y | UN | Y | / | / | / |
| case report | 27(20) | Bindi | 2009 | Recenti Prog med | Y | Y | Y | UN | Y | Y | UN | UN | / | / | / |
| case report | 28(21) | Martínez-Valles | 2009 | Cases J | Y | Y | Y | Y | Y | Y | UN | Y | / | / | / |
| case report | 29(22) | Cicin | 2010 | Trak Univ Tip Fak Derg | Y | Y | Y | Y | Y | Y | UN | Y | / | / | / |
| case report | 32(23) | Suyama | 2011 | Intern Med | Y | Y | Y | Y | Y | Y | UN | Y | / | / | / |
| case report | 33(24) | Stempel | 2013 | BMJ Case Rep | Y | Y | Y | Y | Y | Y | UN | UN | / | / | / |
| case report | 34(25) | Akinosoglou | 2014 | Ann Clin Biochem | Y | Y | Y | Y | Y | Y | UN | Y | / | / | / |
| case report | 36(26) | Nandagopal | 2014 | Am J Ther | Y | Y | Y | Y | Y | N | UN | Y | / | / | / |
| case report | 37(27) | Vega | 2014 | Rev Clin Esp（Barc） | Y | Y | Y | Y | Y | Y | Y | Y | / | / | / |
| case report | 38(28) | Cekerevac | 2015 | Acta Clin Croat | Y | Y | Y | Y | N | N | UN | Y | / | / | / |
| case report | 40(29) | Jeong | 2015 | Tuberc Respir Dis (Seoul) | Y | Y | Y | Y | Y | Y | N | Y | / | / | / |
| case report | 41(30) | Aoki | 2016 | Intern Med | Y | Y | Y | Y | Y | Y | UN | UN | / | / | / |
| case report | 42(31) | Kaya | 2016 | J Clin Diagn Res | Y | Y | Y | Y | Y | Y | UN | Y | / | / | / |
| case report | 43(32) | Ohara | 2016 | Intern Med | Y | Y | Y | Y | Y | Y | UN | Y | / | / | / |
| case report | 44(33) | Hine | 2017 | J Emerg Med | Y | Y | Y | Y | Y | N | UN | Y | / | / | / |
| case report | 45(34) | Wilkins | 2017 | Clin Schizophr Relat Psychoses | Y | Y | Y | Y | Y | Y | N | Y | / | / | / |
| case report | 46(35) | Zhang | 2017 | Thorac Cancer | Y | Y | Y | Y | Y | Y | UN | Y | / | / | / |
| case report | 48(36) | Ferreira | 2018 | BMJ Case Rep | Y | Y | Y | Y | Y | Y | UN | Y | / | / | / |
| case report | 49(37) | Foray | 2018 | Respir Med Case Rep. | Y | Y | Y | Y | Y | Y | UN | Y | / | / | / |
| case report | 51(38) | Kamijo | 2019 | Intern Med | Y | Y | Y | Y | Y | Y | UN | UN | / | / | / |
| case report | 53(39) | Gerhardt | 2020 | Dtsch Med Wochenschr | Y | Y | Y | Y | Y | Y | UN | Y | / | / | / |
| case report | 54(40) | Kosuda | 2020 | Intern Med | Y | Y | Y | Y | Y | Y | UN | Y | / | / | / |
| case report | 55(41) | Cabral | 2020 | Eur J Case Rep Intern Med | Y | Y | Y | Y | Y | Y | UN | Y | / | / | / |
| case report | 56(42) | Pingle | 2020 | ESC Heart Fail | Y | Y | Y | Y | Y | Y | UN | UN | / | / | / |
| case report | 58(43) | Qiang | 2021 | BMC Endocr Disord | Y | Y | Y | Y | Y | Y | Y | Y | / | / | / |
| case report | 59(44) | Senarathne | 2021 | BMJ Case Rep | Y | Y | Y | Y | Y | Y | UN | Y | / | / | / |
| case series | 6(45) | Rieu | 1993 | Horm Res | UN | Y | Y | N | N | Y | Y | Y | UN | Y | / |
| case series | 16(46) | Sakuraba | 2003 | Jpn J Thorac Cardiovasc Surg | Y | Y | Y | Y | Y | Y | Y | Y | UN | UN | / |
| case series | 47(47) | Deldycke | 2018 | Acta Clin Belg | UN | Y | Y | N | N | Y | Y | Y | Y | UN | / |
| case series | 50(48) | Richa | 2018 | Endocrinol Diabetes Metab Case Rep | UN | Y | Y | N | N | Y | Y | Y | UN | UN | / |
| case series | 52(49) | Zhou | 2019 | World J Clin Cases | UN | Y | Y | N | N | Y | Y | Y | UN | UN | / |
| case series | 60(50) | Piasecka | 2022 | Front Med (Lausanne) | Y | Y | Y | Y | Y | Y | Y | Y | Y | Y | / |
| case series | 61(51) | Rosales-Castillo | 2022 | Hipertens Riesgo Vasc | UN | Y | Y | N | N | Y | Y | Y | UN | UN | / |
| retrospective | 3(52) | Dimopoulos | 1992 | Cancer | Y | Y | UN | Y | Y | Y | Y | Y | Y | UN | Y |
| retrospective | 4(53) | Shepherd | 1992 | J Clin Oncol | Y | Y | Y | Y | UN | Y | Y | Y | Y | UN | Y |
| retrospective | 5(54) | Delisle | 1993 | Arch Intern Med | Y | Y | Y | Y | UN | Y | Y | Y | Y | UN | Y |
| retrospective | 10(55) | Winquist | 1995 | J Clin Oncol | Y | Y | Y | Y | UN | Y | Y | Y | Y | UN | Y |
| retrospective | 18(56) | Ilias | 2005 | J Clin Endocrinol Metab | Y | Y | Y | Y | UN | Y | Y | Y | Y | UN | Y |
| retrospective | 30(57) | Doi | 2010 | Endocr J | Y | Y | Y | Y | UN | Y | Y | Y | Y | UN | Y |
| retrospective | 31(58) | Ejaz | 2011 | Cancer | Y | Y | Y | Y | UN | Y | Y | Y | Y | UN | Y |
| retrospective | 35(59) | Nagy-Mignotte | 2014 | J Thorac Oncol | Y | Y | Y | Y | UN | Y | Y | Y | Y | UN | Y |
| prospective | 39(60) | Ghazi | 2015 | Endokrynol Pol | Y | Y | Y | Y | UN | Y | Y | Y | Y | UN | Y |
| retrospective | 57(61) | Lopez-Montoya | 2021 | Arch Endocrinol Metab | Y | Y | UN | Y | UN | Y | Y | Y | Y | UN | Y |

**JBI Critical Appraisal Checklist for Case Reports:** 1. Were patient’s demographic characteristics clearly described? 2. Was the patient’s history clearly described and presented as a timeline? 3. Was the current clinical condition of the patient on presentation clearly described? 4. Were diagnostic tests or assessment methods and the results clearly described? 5. Was the intervention(s) or treatment procedure(s) clearly described? 6. Was the post-intervention clinical condition clearly described? 7. Were adverse events (harms) or unanticipated events identified and described? 8. Does the case report provide takeaway lessons?

**JBI Critical Appraisal Checklist for Case Series:** 1. Were there clear criteria for inclusion in the case series? 2. Was the condition measured in a standard, reliable way for all participants included in the case series ? 3. Were valid methods used for identification of the condition for all participants included in the case series? 4. Did the case series have consecutive inclusion of participants?

5. Did the case series have complete inclusion of participants? 6. Was there clear reporting of the demographics of the participants in the study? 7. Was there clear reporting of clinical information of the participants? 8. Were the outcomes or follow up results of cases clearly reported? 9. Was there clear reporting of the presenting site(s)/clinic(s) demographic information? 10. Was statistical analysis appropriate?

**JBI Critical Appraisal Checklist for Cohort Studies:** 1. Were the two groups similar and recruited from the same population? 2. Were the exposures measured similarly to assign people to both exposed and unexposed groups? 3. Was the exposure measured in a valid and reliable way? 4. Were confounding factors identified? 5. Were strategies to deal with confounding factors stated? 6. Were the groups/participants free of the outcome at the start of the study (or at the moment of exposure)? 7. Were the outcomes measured in a valid and reliable way? 8. Was the follow up time reported and sufficient to be long enough for outcomes to occur? 9. Was follow up complete, and if not, were the reasons to loss to follow up described and explored? 10. Were strategies to address incomplete follow up utilized? 11. Was appropriate statistical analysis used?

**Abbreviations:** JBI, the Joanna Briggs Institute; Y, Yes; N, No; UN, Unclear.

**REFERENCES**

1. Shepherd FA. Ketoconazole. Use in the treatment of ectopic adrenocorticotropic hormone production and Cushing's syndrome in small-cell lung cancer. *Archives of Internal Medicine* (1985) **145**: 863-64. doi:10.1001/archinte.145.5.863

2. Hoffman DM, Brigham B. The use of ketoconazole in ectopic adrenocorticotropic hormone syndrome. *Cancer-Am Cancer Soc* (1991) **67**: 1447-49. doi:10.1002/1097-0142(19910301)67:5<1447::AID-CNCR2820670531>3.0.CO;2-I

3. Tabata M, Ohnoshi T, Ueoka H, Kiura K, Segawa Y, Shibayama T, Maeda T, Miyatake K, Takigawa N, Kimura I. [A case of small cell lung cancer associated with diabetes insipidus and Cushing's syndrome]. *Nihon Kyobu Shikkan Gakkai Zasshi* (1993) **31**: 235-39.

4. Auchus RJ, Mastorakos G, Friedman TC, Chrousos GP. Corticotropin-releasing hormone production by a small cell carcinoma in a patient with ACTH-dependent Cushing’s syndrome. *J Endocrinol Invest* (1994) **17**: 447-52. doi:10.1007/BF03347737

5. Huang TP, Wang PW, Liu RT, Tung SC, Jean WY, Lu YC, Hung CL, Wu CC, Chen WJ. Ectopic ACTH syndrome with nocardiosis--a case report. *Changgeng Yi Xue Za Zhi* (1994) **17**: 371-77.

6. Takano K, Takayama K, Nakano H, Hagimoto N, Nakanishi Y, Hara N. Small cell lung cancer associated with ectopic ACTH syndrome. *Nihon Kyōbu Shikkan Gakkai zasshi* (1996) **34**: 220-25.

7. Sato S, Yokoyama A, Ohtsuka T, Nomoto T, Abe M, Kohno N, Hiwada K. Cushing's Syndrome due to Small Cell Lung Cancer with Ectopic Production of Adrenocorticotropic and Parathyroid Hormone. *Nippon Ronen Igakkai Zasshi. Japanese Journal of Geriatrics* (1997) **34**: 215-20. doi:10.3143/geriatrics.34.215

8. Castro Cabezas M, Vrinten DH, Burgers JA, Croughs RJ. Central diabetes insipidus and Cushing's syndrome due to ectopic ACTH production by disseminated small cell lung cancer: a case report. *Neth J Med* (1998) **53**: 32.

9. Bodvarsson S, Burlingham W, Kusaka S, Hafez GR, Becker BN, Pintar T, Sollinger HW, Albertini MR. Donor-derived small cell lung carcinoma in a kidney transplant recipient. *Cancer-Am Cancer Soc* (2001) **92**: 2429-34. doi:10.1002/1097-0142(20011101)92:9<2429::aid-cncr1592>3.0.co;2-g

10. Dube L, Daenen S, Kouatchet A, Soltner C, Alquier P. [Severe metabolic alkalosis following hypokalemia from a paraneoplastic Cushing syndrome]. *Ann Fr Anesth Reanim* (2001) **20**: 860-64. doi:10.1016/s0750-7658(01)00518-4

11. Agha A, Brennan S, Moore KB, Grogan L, Thompson CJ. Small-cell lung cancer presenting as diabetes insipidus and Cushing's syndrome. *Pituitary* (2005) **8**: 105-07. doi:10.1007/s11102-005-3308-1

12. Hadem J, Cornberg M, Länger F, Schedel I, Kirchhoff T, Niedermeyer J, Manns MP, Schöfl C. Making sense of muscle fatigue and liver lesions. *Zeitschrift für Gastroenterologie* (2007) **45**: 609.

13. Lee H, 양홍준, 성혜정, 김지은, 박진민, 박찬권, 노은숙, 조재형, 고승현, 송기호 et al. A Case of Ectopic ACTH Syndrome Associated with Small Cell Lung Cancer Presented with Hypokalemia (2007) **22**: 359-64.

14. Müssig K, Maser-Gluth C, Hartmann M, Wehrmann M, Horger M, Kanz L, Häring HU, Wudy SA. 68-year-old female patient with dyspnea and hypokalemic hypertension. *Der Internist* (2007) **48**: 1145-50. doi:10.1007/s00108-007-1930-x

15. Servonnet A, Delacour H, Roux X, Dehan C, Gardet V, Morand C. Ectopic ACTH syndrome and severe hypokalaemia. *Annales de biologie clinique (Paris)* (2007) **65**: 425. doi:10.1684/abc.2007.0142

16. Tanaka H, Kobayashi A, Bando M, Hosono T, Tsujita A, Yamasawa H, Ohno S, Hironaka M, Sugiyama Y. [Case of small cell lung cancer complicated with diabetes insipidus and Cushing syndrome due to ectopic adrenocorticotropic hormone secretion]. *Nihon Kokyuki Gakkai Zasshi* (2007) **45**: 793-98.

17. Fernandez-Rodriguez E, Villar-Taibo R, Pinal-Osorio I, Cabezas-Agricola JM, Anido-Herranz U, Prieto A, Casanueva FF, Araujo-Vilar D. Severe hypertension and hypokalemia as first clinical manifestations in ectopic Cushing's syndrome. *Arq Bras Endocrinol Metabol* (2008) **52**: 1066-70. doi:10.1590/s0004-27302008000600019

18. Guabello G, Brunetti L, Palladini G, Musumeci S, Lovati E, Perfetti V. Paraneoplastic Cushing’s Syndrome and Nephrotic Syndrome in a Patient With Disseminated Small Cell Lung Cancer. *American Journal of Clinical Oncology* (2008) **31**: 102-03. doi:10.1097/01.coc.0000203741.06225.8a

19. Vadlamudi RS, Van Dort M, Barklow T, Byrd RJ, Moorman JP. Strongyloides hyperinfection syndrome complicating (ectopic) Cushing syndrome. *South Med J* (2008) **101**: 750-52. doi:10.1097/SMJ.0b013e31817a836e

20. Bindi M, Moruzzo D, Pinelli M, Rosada J, Castiglioni M. [Hypokalemia from ectopic ACTH secretion and hypothiroidism in patient affected by small cell lung cancer]. *Recenti Prog Med* (2009) **100**: 137-39.

21. Martínez-Valles MA, Palafox-Cazarez A, Paredes-Avina JA. Severe hypokalemia, metabolic alkalosis and hypertension in a 54 year old male with ectopic ACTH syndrome: a case report. *Cases Journal* (2009) **2**: 6174. doi:10.4076/1757-1626-2-6174

22. Cicin I, Uzunoglu S, Ermantas N, Usta U, Temizoz O, Karagol H. A Destroyer Immunologic Cause In Small Cell Lung Carcinoma: Ecthopic Cushing's Syndrome. *Medical Journal of Trakya University* (2010) **27**: 312-14. doi:10.5174/tutfd.2008.00718.1

23. Suyama K, Naito Y, Yoh K, Niho S, Goto K, Ohmatsu H, Nishiwaki Y, Ohe Y. Development of Cushing's syndrome during effective chemotherapy for small cell lung cancer. *Intern Med* (2011) **50**: 335-38. doi:10.2169/internalmedicine.50.4127

24. von Stempel C, Perks C, Corcoran J, Grayez J. Cardio-respiratory failure secondary to ectopic Cushing's syndrome as the index presentation of small-cell lung cancer. *Case Reports* (2013) **2013**: r2013009974. doi:10.1136/bcr-2013-009974

25. Akinosoglou K, Siagris D, Geropoulou E, Kosmopoulou O, Velissaris D, Kyriazopoulou V, Gogos C. Hyperamylasaemia and dual paraneoplastic syndromes in small cell lung cancer. *Annals of Clinical Biochemistry: International Journal of Laboratory Medicine* (2014) **51**: 101-05. doi:10.1177/0004563213500658

26. Nandagopal L, Arias C, Pillai U, Osman-Malik Y. Ectopic ACTH and Cisplatin Toxicity—A Diagnostic Dilemma. *Am J Ther* (2014) **21**: e154-56. doi:10.1097/MJT.0b013e3182691b03

27. Pérez Vega C. Panhipopituitarismo reversible en un paciente con síndrome de Cushing por secreción ectópica de hormona adrenocorticotropa secundaria a un carcinoma microcítico de pulmón. *Revista Clínica Española* (2014) **214**: e5-08. doi:10.1016/j.rce.2013.10.007

28. Cekerevac I, Petrović M, Novković L, Bubanja D, Bubanja I, Djokić B, Stanković V, Jurisić V. ECTOPIC ACTH SECRETION WITH CONCOMITANT HYPERAMYLASEMIA IN A PATIENT WITH SMALL CELL LUNG CARCINOMA: CASE REPORT. *Acta clinica Croatica (Tisak)* (2015) **54**: 536-40.

29. Jeong C, Lee J, Ryu S, Lee HY, Shin AY, Kim JS, Ahn JH, Kang HS. A Case of Ectopic Adrenocorticotropic Hormone Syndrome in Small Cell Lung Cancer. *Tuberculosis and respiratory diseases* (2015) **78**: 436-39. doi:10.4046/trd.2015.78.4.436

30. Aoki M, Fujisaka Y, Tokioka S, Hirai A, Henmi Y, Inoue Y, Narabayashi K, Yamano T, Tamura Y, Egashira Y et al. Small-cell Lung Cancer in a Young Adult Nonsmoking Patient with Ectopic Adrenocorticotropin (ACTH) Production. *Internal medicine (Tokyo, 1992)* (2016) **55**: 1337-39. doi:10.2169/internalmedicine.55.6139

31. Kaya T. Severe Hypokalaemia, Hypertension, and Intestinal Perforation in Ectopic Adrenocorticotropic Hormone Syndrome. *JOURNAL OF CLINICAL AND DIAGNOSTIC RESEARCH* (2016). doi:10.7860/JCDR/2016/17198.7127

32. Ohara N, Kaneko M, Sato K, Usuda H, Tanaka J, Maekawa T, Sasano H, Katakami H, Kaneko K, Kamoi K. Acute Exacerbation of Idiopathic Pulmonary Fibrosis Following Treatment for Cushing's Syndrome. *Internal Med* (2016) **55**: 389-94. doi:10.2169/internalmedicine.55.5566

33. Hine J, Schwell A, Kairys N. An Unlikely Cause of Hypokalemia. *The Journal of Emergency Medicine* (2017) **52**: e187-91. doi:10.1016/j.jemermed.2016.12.011

34. Wilkins CM, Johnson VL, Fargason RE, Birur B. Psychosis as a sequelae of paraneoplastic syndrome in Small- Cell Lung Carcinoma: A psycho-neuroendocrine interface. *Clinical Schizophrenia & Related Psychoses* (2017). doi:10.3371/CSRP.CWVJ.111717

35. Zhang HY, Zhao J. Ectopic Cushing syndrome in small cell lung cancer: A case report and literature review. *Thorac Cancer* (2017) **8**: 114-17. doi:10.1111/1759-7714.12403

36. Lobo Ferreira T, Nunes Da Silva T, Canário D, Francisca Delerue M. Hypertension and severe hypokalaemia associated with ectopic ACTH production. *BMJ Case Reports* (2018): 2017-223406. doi:10.1136/bcr-2017-223406

37. Foray N, Stone T, Johnson A, Ali M, Kulkarni S, Gao J, Sreedhar R. Severe metabolic alkalosis–a diagnostic dilemma. *Respiratory Medicine Case Reports* (2018) **25**: 177-80. doi:10.1016/j.rmcr.2018.08.019

38. Kamijo S, Hasuike S, Nakamura K, Takaishi Y, Yamada Y, Ozono Y, Tsuchimochi M, Sueta M, Kusumoto K, Iwakiri H et al. Acute Liver Failure Due to Severe Hepatic Metastasis of Small-cell Lung Cancer Producing Adrenocorticotropic Hormone Complicating Ectopic Cushing Syndrome. *Internal Med* (2019) **58**: 2977-82. doi:10.2169/internalmedicine.1976-18

39. Gerhardt LMS, Sabath L, Müller B, Capraro J, Borm K. Paraneoplastisches Cushing-Syndrom als Ursache von therapierefraktärer Hypokaliämie. *DMW - Deutsche Medizinische Wochenschrift* (2020) **145**: 783-86. doi:10.1055/a-1163-9873

40. Kosuda A, Shirahata T, Kudo N, Uehara Y, Miyawaki M, Hagiwara A, Murakami R, Shimizu K. Long-term Survival of a Patient with Small Cell Lung Cancer Secreting ADH and ACTH Simultaneously, Following the Prolonged Use of Amrubicin. *Internal Med* (2020) **59**: 107-12. doi:10.2169/internalmedicine.2838-19

41. Lemos CS, Deveza N, Baptista JP, Martins P. Disseminated Strongyloides stercoralis Infection Associated with Endogenous Hypercortisolism - A Case Report. *Eur J Case Rep Intern Med* (2020) **7**: 1509. doi:10.12890/2020_001509

42. Pingle SR, Shah T, Mosleh W, Kim AS. Cushing syndrome cardiomyopathy: an unusual manifestation of small‐cell lung cancer. *ESC Heart Failure* (2020) **7**: 3189-92. doi:10.1002/ehf2.12860

43. Qiang W, Song S, Chen T, Wang Z, Feng J, Zhang J, Guo H. A rare case of ectopic ACTH syndrome with rhabdomyolysis. *Bmc Endocr Disord* (2021) **21**. doi:10.1186/s12902-021-00755-0

44. Senarathne UD, Dayanath BKTP, Punchihewa R, Gunasena B. Patient with respiratory distress, facial oedema and refractory hypokalaemia. *BMJ Case Reports* (2021) **14**: e240330. doi:10.1136/bcr-2020-240330

45. Rieu M, Rosilio M, Richard A, Vannetzel J, Kuhn J. Paradoxical Effect of Somatostatin Analogues on the Ectopic Secretion of Corticotropin in Two Cases of Small Cell Lung Carcinoma. *Hormone Research* (1993) **39**: 207-12. doi:10.1159/000182737

46. Sakuraba M, Murasugi M, Oyama K, Adachi T, Ikeda T, Onuki T. Diagnosis and surgical treatment of ectopic adrenocorticotropic hormone-producing pulmonary tumors accompanied by Cushing syndrome. *The Japanese Journal of Thoracic and Cardiovascular Surgery* (2003) **51**: 656-59. doi:10.1007/s11748-003-0004-9

47. Deldycke A, Haenebalcke C, Taes Y. Paraneoplastic Cushing syndrome, case-series and review of the literature. *Acta clinica belgica (English ed. Online)* (2018) **73**: 298-304. doi:10.1080/17843286.2017.1373927

48. Richa CG, Saad KJ, Halabi GH, Gharios EM, Nasr FL, Merheb MT. Case-series of paraneoplastic Cushing syndrome in small-cell lung cancer. *Endocrinology, Diabetes & Metabolism Case Reports* (2018) **2018**. doi:10.1530/EDM-18-0004

49. Zhou T, Wang Y, Zhao X, Liu Y, Wang YX, Gang XK, Wang GX. Small cell lung cancer starting with diabetes mellitus: Two case reports and literature review. *World J Clin Cases* (2019) **7**: 1213-20. doi:10.12998/wjcc.v7.i10.1213

50. Piasecka M, Larsson M, Papakokkinou E, Olsson L, Ragnarsson O. Is ectopic Cushing’s syndrome underdiagnosed in patients with small cell lung cancer? *Frontiers in Medicine* (2022) **9**. doi:10.3389/fmed.2022.954033

51. Rosales-Castillo A, Bustos-Merlo A. Arterial hypertension of infrequent cause. *Hipertensión y Riesgo Vascular* (2022) **39**: 92-94. doi:10.1016/j.hipert.2021.09.002

52. Dimopoulos MA, Fernandez JF, Samaan NA, Holoye PY, Vassilopoulou-Sellin R. Paraneoplastic Cushing's syndrome as an adverse prognostic factor in patients who die early with small cell lung cancer. *Cancer-Am Cancer Soc* (1992) **69**: 66-71. doi:10.1002/1097-0142(19920101)69:1<66::aid-cncr2820690113>3.0.co;2-2

53. Shepherd FA, Laskey J, Evans WK, Goss PE, Johansen E, Khamsi F. Cushing's syndrome associated with ectopic corticotropin production and small-cell lung cancer. *J Clin Oncol* (1992) **10**: 21.

54. Delisle L, Boyer MJ, Warr D, Killinger D, Payne D, Yeoh JL, Feld R. Ectopic corticotropin syndrome and small-cell carcinoma of the lung. Clinical features, outcome, and complications. *Arch Intern Med* (1993) **153**: 746-52.

55. Winquist EW, Laskey J, Crump M, Khamsi F, Shepherd FA. Ketoconazole in the management of paraneoplastic Cushing's syndrome secondary to ectopic adrenocorticotropin production. *J Clin Oncol* (1995) **13**: 157-64. doi:10.1200/JCO.1995.13.1.157

56. Ilias I, Torpy DJ, Pacak K, Mullen N, Wesley RA, Nieman LK. Cushing’s Syndrome Due to Ectopic Corticotropin Secretion: Twenty Years’ Experience at the National Institutes of Health. *The Journal of Clinical Endocrinology & Metabolism* (2005) **90**: 4955-62. doi:10.1210/jc.2004-2527

57. Doi M, Sugiyama T, Izumiyama H, Yoshimoto T, Hirata Y. Clinical features and management of ectopic ACTH syndrome at a single institute in Japan. *Endocr J* (2010) **57**: 1061-69. doi:10.1507/endocrj.K10E-265

58. Ejaz S, Vassilopoulou-Sellin R, Busaidy NL, Hu MI, Waguespack SG, Jimenez C, Ying AK, Cabanillas M, Abbara M, Habra MA. Cushing syndrome secondary to ectopic adrenocorticotropic hormone secretion. *Cancer-Am Cancer Soc* (2011) **117**: 4381-89. doi:10.1002/cncr.26029

59. Nagy-Mignotte H, Shestaeva O, Vignoud L, Guillem P, Ruckly S, Chabre O, Sakhri L, Duruisseaux M, Mousseau M, Timsit JF et al. Prognostic impact of paraneoplastic cushing's syndrome in small-cell lung cancer. *J Thorac Oncol* (2014) **9**: 497-505. doi:10.1097/JTO.0000000000000116

60. Ghazi AA, Abbasi Dezfooli A, Amirbaigloo A, Daneshvar Kakhki A, Mohammadi F, Tirgari F, Pourafkari M. Ektopowy zespół Cushinga u pacjentów z nowotworem śródpiersia lub płuc — doniesienie z ośrodka trzeciego stopnia referencyjności w Iranie. *Endokrynol Pol* (2015) **66**: 2-09. doi:10.5603/EP.2015.0002

61. Lopez-Montoya V, Gutierrez-Restrepo J, Grajales JLT, Aristizabal N, Pantoja D, Roman-Gonzalez A, Jimenez C. Ectopic Cushing syndrome in Colombia. *Archives of Endocrinology and Metabolism* (2020). doi:10.20945/2359-3997000000271

| **Supplementary Table 2.** FDA Drug Approval Notifications for SCLC. | | | | |
| --- | --- | --- | --- | --- |
| **Date** | **Approval** | **Description** | **Trials** | **Withdrawn** |
| 2020-06-15 | Accelerated approval to lurbinectedin for metastatic SCLC. | Food and Drug Administration granted accelerated approval to lurbinectedin(ZEPZELCA, Pharma Mar S.A.) for adult patients with metastatic small cell lung cancer (SCLC) with disease progression on or after platinum-based chemotherapy. | PM1183-B-005-14 trial  (Study B-005; NCT02454972) |  |
| 2020-03-30 | Approved durvalumab for ES-SCLC. | Food and Drug Administration approved durvalumab (IMFINZI, AstraZeneca) in combination with etoposide and either carboplatin or cisplatin as first-line treatment of patients with extensive-stage small cell lung cancer (ES-SCLC). | CASPIAN (NCT03043872) |  |
| 2019-06-17 | Accelerated approval to pembrolizumab for metastatic SCLC. | Food and Drug Administration granted accelerated approval to pembrolizumab (KEYTRUDA, Merck) for patients with metastatic small cell lung cancer (SCLC) with disease progression on or after platinum-based chemotherapy and at least one other prior line of therapy. | KEYNOTE-158 (NCT02628067)；  KEYNOTE-028 (NCT02054806) | 2021/3/30, KEYNOTE-604 |
| 2019-03-18 | Approved atezolizumab ES-SCLC. | Food and Drug Administration approved atezolizumab (TECENTRIQ, Genentech Inc.) in combination with carboplatin and etoposide, for the first-line treatment of adult patients with extensive-stage small cell lung cancer (ES-SCLC). | IMpower133 (NCT02763579) |  |
| 2018-08-16 | Accelerated approval to nivolumab for metastatic SCLC. | Food and Drug Administration granted accelerated approval to nivolumab (Opdivo, Bristol-Myers Squibb Company Inc.) for patients with metastatic small cell lung cancer (SCLC) with progression after platinum-based chemotherapy and at least one other line of therapy. | CheckMate-032 (NCT01928394) | 2020/12/29, CheckMate 331 |
